# Lymph node fine-tuning FcγR signaling boosts anti-PD-1 therapy

**DOI:** 10.1136/jitc-2025-014665

**Published:** 2026-06-12

**Authors:** Marion V Guérin, Mathilde Ruggiu, Lea C Feldmann, Béatrice Corre, Pauline Ombredanne, Bruno Iannascoli, Zacarias Garcia, Fabrice Lemaître, Lynn Macdonald, Pierre Guermonprez, Capucine L Grandjean, Pierre Bruhns, Philippe Bousso

**Affiliations:** 1 INSERM U1223, Université Paris Cité, Immunology, Dynamics of Immune Responses, Institut Pasteur, Paris, France; 2 INSERM UMR1222, Université Paris Cité, Immunology, Antibodies in Therapy and Pathology, Institut Pasteur, Paris, France; 3 Regeneron Pharmaceuticals Inc, Tarrytown, New York, USA; 4 Immunology, Institut Pasteur, Paris, France

**Keywords:** Immune Checkpoint Inhibitor, T cell, Clonality, Solid tumor, Monoclonal antibody

## Abstract

**Background:**

Anti-PD-1 monoclonal antibody (mAb) therapy promotes the emergence of new T cell clonotypes within tumors, suggesting de novo priming in the periphery. Yet, the mechanisms that mobilize these additional T cells remain poorly defined.

**Methods:**

We investigated the impact of anti-PD-1 mAbs on circulating T cell dynamics and their recruitment into tumor-draining lymph nodes (TDLNs) using multiple transgenic mouse models, including FcγR-deficient and type I interferon receptor-deficient mice. To dissect the role of FcγR engagement, we compared an Fc-silent anti-PD-1 LALAPG variant alongside conventional anti-PD-1 antibodies, and further extended our study to an additional immune checkpoint inhibitor for comparison. These approaches were complemented by experiments in FcγR-humanized mice using a human IgG4 anti-PD-1 mAb. In parallel, the effects of the therapeutic IgG4 antibody nivolumab were evaluated in human cell-based assays using dynamic imaging.

**Results:**

Here, we demonstrate that anti-PD-1 mAbs promote the recruitment of circulating T cells into TDLNs, resulting in an expanded anti-tumor T cell response. This influx was part of a general reactive lymphadenopathy that required FcγR engagement and type I IFN production, leading to a burst of chemokine release. These results were extended to FcgR-humanized mice treated with a human IgG4 variant of the anti-PD-1 mAb and were similarly observed with another immune checkpoint inhibitor, anti-TIM-3, broadening this mechanism as a major one during checkpoint blockade in the LN.

**Conclusions:**

Our results reveal a previously unrecognized role for low to moderate FcγR engagement in TDLNs, which amplifies the anti-tumor T-cell response elicited during anti-PD-1 therapy.

**WHAT IS ALREADY KNOWN ON THIS TOPIC**

- Anti-PD-1 monoclonal antibody (mAb) therapy enhances anti-tumor immunity and is associated with the emergence of new T cell clonotypes within tumors, suggesting de novo priming in the periphery. However, the mechanisms driving the recruitment and mobilization of these additional T cells remain poorly understood.

**WHAT THIS STUDY ADDS**

- This study demonstrates that anti-PD-1 therapy promotes the recruitment of circulating T cells into tumor-draining lymph nodes, leading to an expanded anti-tumor response. It identifies a previously unrecognized mechanism involving FcγR engagement and type I interferon signaling, which induces reactive lymphadenopathy and chemokine release. Furthermore, it shows that low to moderate FcγR engagement can bridge FcγR-expressing cells and PD-1^+^ T cells to drive chemokine production without inducing phagocytosis. These findings were validated with a human IgG4 anti-PD-1 mAb and extended to another immune checkpoint inhibitor, anti-TIM-3.

**HOW THIS STUDY MIGHT AFFECT RESEARCH, PRACTICE OR POLICY**

- These results highlight FcγR engagement as a key modulator of immune checkpoint blockade efficacy and suggest that fine-tuning Fc-FcγR interactions could enhance therapeutic responses. This may inform the design of next-generation checkpoint inhibitors and antibody engineering strategies, as well as guide combination therapies aimed at improving T cell recruitment and anti-tumor immunity.

## Introduction

 Anti-PD-1 therapy promotes clinical responses in a variety of cancers by reinvigorating and expanding intratumoral CD8^+^ T cell responses. A major cellular target of anti-PD-1 monoclonal antibody (mAb) are tumor-specific T cells with stem-like characteristics often referred to as progenitor exhausted T cells. These T cells, which express the transcription factor TCF-1, preferentially expand during PD-1 blockade, and their presence in the tumor microenvironment is associated with good clinical outcomes.^1^,^8^

In addition to expanding pre-existing tumor-specific T cells, anti-PD-1 therapy has been shown to promote the recruitment of new T cell clonotypes at the tumor site. Indeed, comparison of pre-treatment and post-treatment tumor biopsies has provided evidence for clonal renewal or clonal replacement.^9^,^12^ The emergence of new T cell clonotypes during anti-PD-1 therapy suggests de novo T cell priming in secondary lymphoid organs. The importance of a peripheral mode of action is further supported by the observation that T cell proliferation in the blood after PD-1 blockade is predictive of clinical responses.^12^ In fact, recent studies have demonstrated an important role for lymph nodes during anti-PD-1 treatment. First, the tumor-draining lymph node was found to be a major reservoir of stem-like precursor exhausted T cells.^13^,^15^ Second, surgical resection of tumor-draining lymph nodes substantially reduced anti-PD-1 mAb-induced anti-tumor activity in mice.^16^ Similarly, blocking T cell egress from lymph nodes using the sphingosine 1-phosphate receptor agonist FTY720 after anti-PD-1 mAb administration diminishes anti-tumor activity in preclinical models.^10 17^ Conversely, targeting the delivery of anti-PD-1 mAb to the draining lymph node promoted anti-tumor responses.^18^ We recently identified a mechanism operating in the lymph node whereby anti-PD-1 mAbs bind to follicular helper T cells (Tfh), stimulating production of IL-4, and thereby enhancing the local proliferation of antigen-specific CD8^+^ T cells.^17^ While Tfh-derived IL-4 increased the progeny of existing antigen-specific CD8^+^ T cells in the tumor-draining lymph node, it may not fully explain the emergence of new T cell clones. Therefore, how anti-PD-1 mAbs may broaden the clonal composition of tumor-specific T cells remains to be fully understood.

Here, we uncovered a new mechanism by which anti-PD-1 mAbs promote the recruitment of circulating T cells in lymph nodes. Such recruitment was part of a general reactive lymphadenopathy, which required both FcγR engagement and type I IFN signaling and was associated with a burst of chemokine release. In mice with humanized FcγRs, a human IgG4 swap version of the murine anti-PD-1 mAb also promoted immune cell recruitment in lymph nodes. Finally, we demonstrated that the mobilization of circulating T cells in the lymph node substantially contributed to the anti-tumor activity of anti-PD-1 mAbs.

## Results

### Anti-PD-1 therapy promotes both T cell recruitment and proliferation in the tumor-draining lymph node

**Figure 1:**
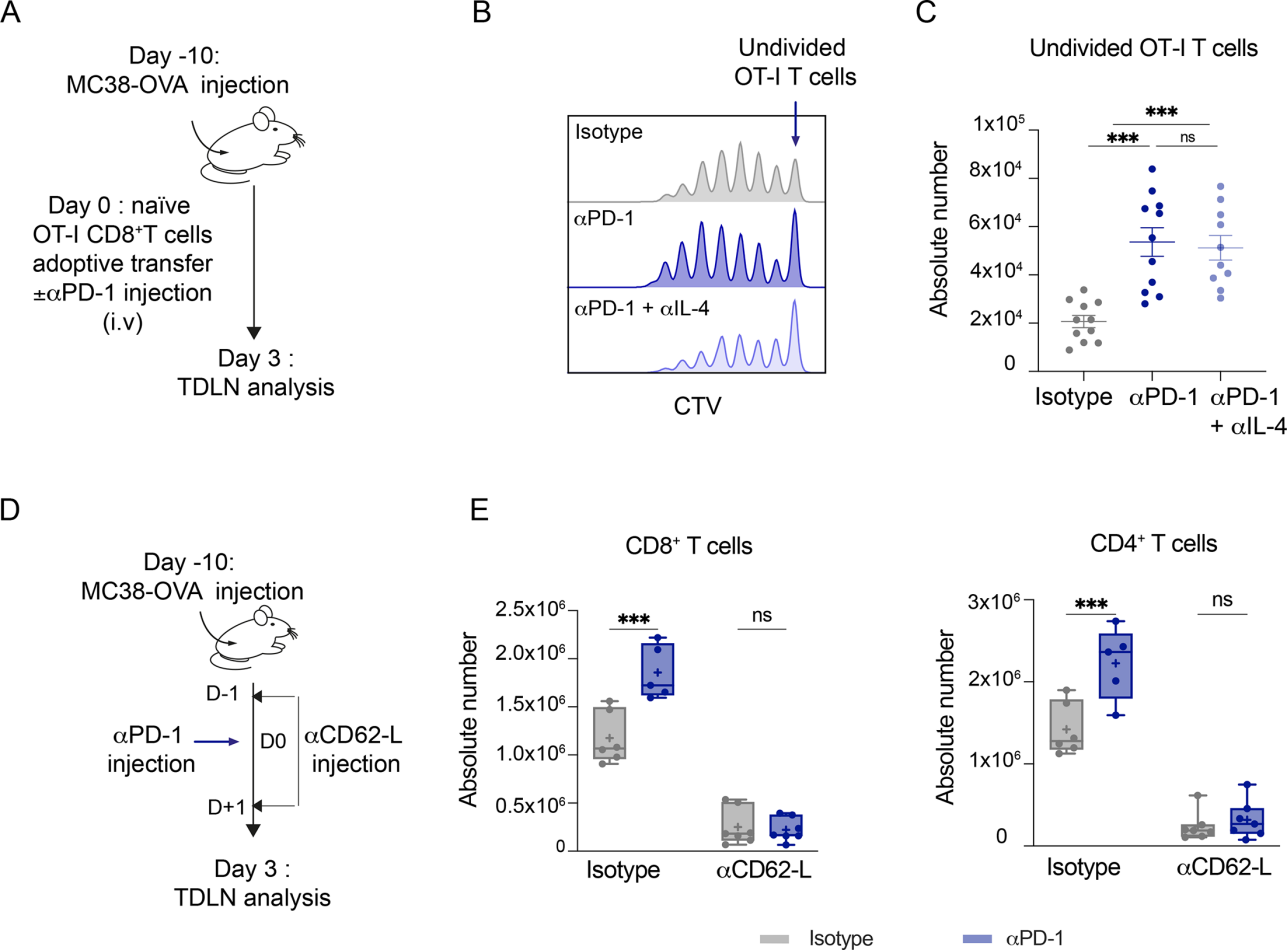
Anti-PD-1 therapy promotes T cell accumulation in draining lymph nodes. (**A–E**) MC38-OVA tumor-bearing mice were adoptively transferred with naïve CTV-labeled OT-I CD8^+^ T cells on day 10 and treated with anti-PD-1 mAb alone, anti-PD-1 mAb in combination with an anti-IL-4 mAb, or with isotype control. OT-I CD8^+^ T cell proliferation in the draining lymph node was assessed on day 3 post-treatment. (**A**) Experimental set-up. (**B**) Representative histograms showing CTV dilution in OT-I CD8^+^ T cells. (**C**) Absolute number of undivided OT-I CD8^+^ T cells in lymph nodes from mice bearing MC38-OVA. Compiled from three independent experiments with a total of 10–12 mice per group. (**D, E**) MC38-OVA tumor-bearing mice were treated or not with anti-CD62-L to block lymphocyte entry into the lymph nodes, followed by treatment with anti-PD-1 mAb or isotype control on day 10. (**D**) Experimental set-up. (**E**) Quantification of CD8^+^ T cells and CD4^+^ T cells in TDLNs 3 days after anti-PD-1 mAb treatment. Compiled from two independent experiments with a total of six mice per group. Statistical analyses were performed using Kruskal-Wallis one-way ANOVA (**C, E**). ns, non-significant; ***p<0.001. ANOVA, analysis of variance; CTV, CellTrace Violet; i.v., intravenous; mAb, monoclonal antibody; TDLN, tumor-draining lymph nodes.

Beyond their role at the tumor site, anti-PD-1 mAbs act in lymphoid organs to increase anti-tumor T cell responses. We have previously reported that anti-PD-1 mAb elicits IL-4 driven T cell proliferation in tumor-draining lymph nodes by binding to and activating Tfh cells.^17^ While this mechanism enhances the magnitude of existing T cell responses, it may not necessarily explain the T cell clonal renewal seen in patient treated with anti-PD-1 mAb.^9^,^12^ We therefore sought to determine whether additional mechanisms contributed to the peripheral activity of anti-PD-1 mAb. When analyzing antigen-specific CD8^+^ T cells in tumor-draining lymph nodes on anti-PD-1 mAb treatment, we observed that, concomitant with enhanced proliferation, there was an increase in the number of undivided CD8^+^ T cells, most likely reflecting increased recruitment (figure 1A–C). Interestingly, when IL-4 was blocked during anti-PD-1 therapy, the boost in T cell proliferation was abrogated, but the mobilization of undivided T cells was maintained (figure 1B,C). To confirm that anti-PD-1 mAb promotes T cell recruitment in the lymph node, we blocked CD62L, which is essential for naïve T cell entry (figure 1D). In this setting, both CD8^+^ and CD4^+^ T cells accumulated in the lymph node on anti-PD-1 mAb administration, and these effects were lost on CD62L blockade (figure 1E). These data suggest that anti-PD-1 therapy promotes T cell recruitment in the tumor-draining lymph node, independently of its effects on T cell proliferation.

### Anti-PD-1 treatment induces swelling of the tumor-draining lymph node

**Figure 2:**
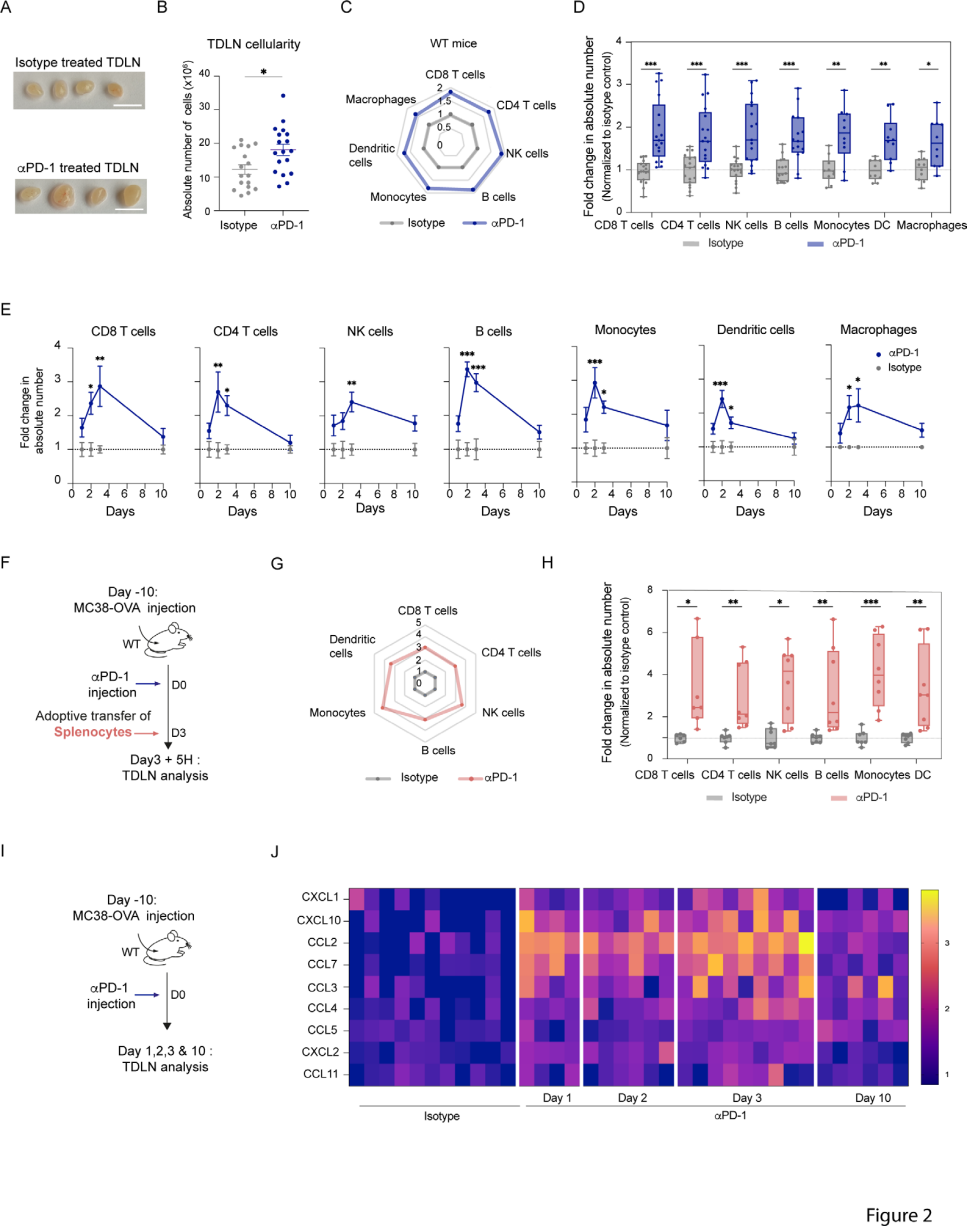
Anti-PD-1 therapy triggers lymph node swelling associated with a burst of chemokine production. (**A, B**) MC38-OVA tumor-bearing mice were treated on day 10 with anti-PD-1 mAb or with isotype control. (**A**) Representative tumor-draining lymph nodes (TDLNs) from mice treated or not with anti-PD-1 mAb. Scale bar 1 cm. (**B**) Absolute numbers of cells present in TDLNs assessed 3 days after anti-PD-1 mAb treatment. Compiled from four independent experiments with a total of 17–18 mice per group. (**C, D**) Quantification of immune cells was performed 3 days later by flow cytometry in TDLNs, represented as a spider chart (**C**) and fold changes (**D**) in absolute number compared with the isotype control group. Compiled from four independent experiments with a total of 16–18 mice per group. (**E**) Quantification of immune cells in TDLNs on day 1, day 2, day 3, and day 10 after anti-PD-1 mAb treatment, expressed as a fold change compared with the isotype control group. Compiled from three independent experiments with a total of 6–10 mice per group for each time point. (**F–H**) MC38-OVA tumor-bearing mice were treated on day 10 with anti-PD-1 mAb or with isotype control. 3 days later, GFP^+^ splenocytes were adoptively transferred, and their recruitment to the TDLNs was evaluated 5 hours after the transfer. (**F**) Experimental set-up. (**G, H**) Quantification of immune cells by flow cytometry in TDLNs, represented as a spider chart (**G**) and fold changes (**H**) in absolute number compared with the isotype control group. Compiled from two independent experiments with a total of 6–8 mice per group. (**I, J**) MC38-OVA tumor-bearing mice were treated on day 10 with anti-PD-1 mAb or with isotype control. TDLN lysates were collected on day 1, 2, 3, and 10 post-treatment. (**I**) Experimental set-up. (**J**) Heat map showing the cytokine landscape as detected by multiplex protein assay of the draining lymph nodes. Values were normalized to the mean value measured for control isotype-treated mice. Compiled from two independent experiments with a total of 4–10 mice per group. Statistical analyses were performed using Mann-Whitney U t-tests (**B, E**) or Kruskal-Wallis one-way ANOVA (**D, H**). *p<0.05; **p<0.01; ***p<0.001. ANOVA, analysis of variance; DC, dendritic cell; mAb, monoclonal antibody.

When analyzing mice bearing an MC38-OVA tumor, we noted that anti-PD-1 mAb treatment resulted in a substantial and rapid increase in lymph node size and cellularity (figure 2A,B).

To test whether lymph node enlargement was solely due to the observed T cell accumulation, we measured the absolute number of various immune cell types in tumor-draining lymph nodes. As shown in figure 2C,D, anti-PD-1 mAb treatment resulted in the accumulation of all immune cells tested, including T cells, B cells, NK cells, monocytes, macrophages, and dendritic cells (DCs). These cellular changes occurred within 1–2 days after anti-PD-1 mAb injection, with a residual increase in immune cell numbers still maintained over time (figure 2E). To extend these results, tumor-bearing mice were injected with anti-PD-1 mAb and 3 days later, adoptively transferred with GFP-expressing splenocytes to assess their homing to lymph nodes within the next few hours (figure 2F). Lymph nodes of anti-PD-1 mAb treated mice accumulated more GFP-positive cells than isotype treated mice within this short period of time (figure 2G,H). Thus, anti-PD-1 mAb treatment modifies the lymph node microenvironment, favoring the recruitment of additional immune cells.

### Anti-PD-1 mAbs trigger a burst of chemokines in the tumor-draining lymph node

To evaluate changes in the lymph node microenvironment on anti-PD-1 mAb treatment, we measured the concentration of various immune cell-attracting chemokines (figure 2I). As shown in figure 2J, the levels of multiple chemokines were elevated early after anti-PD-1 mAb administration, including CCL2, CCL3, CCL4, CCL5, CCL8, CCL11, CXCL1, CXCL2, and CXCL10. These increases were mostly due to local production in the lymph node and did not depend on circulating chemokines since their serum levels remain unchanged (online supplemental figure S1). Altogether, our results indicate that anti-PD-1 therapy strongly modifies the lymph node environment, increasing chemokine levels and promoting the recruitment of multiple types of immune cells.

### FcγRIII engagement is essential for immune cell mobilization in the tumor-draining lymph node during anti-PD-1 therapy

**Figure 3:**
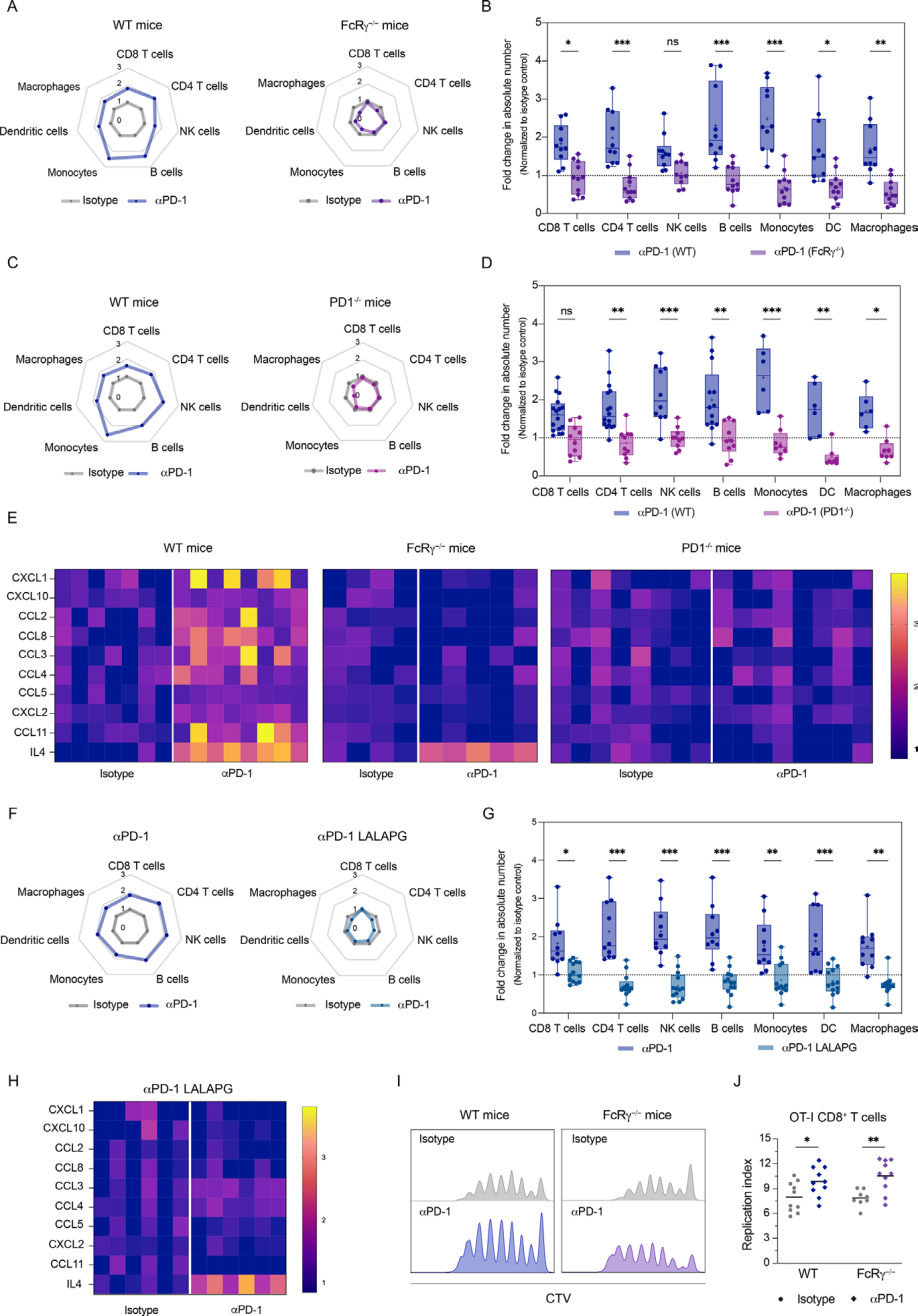
FcγR engagement is critical for immune cell recruitment within the tumor-draining lymph node during anti-PD-1 therapy. MC38-OVA tumor-bearing mice were treated on day 10 with anti-PD-1 mAb or with isotype control. (**A, B**) Quantification of immune cells in WT and FcRγ^−/−^ mice, represented as (**A**) a spider chart and (**B**) fold change in absolute number normalized to each isotype control group. Compiled from three independent experiments with a total of 10–12 mice per group. Quantification of immune cells in WT and PD-1^−/−^ mice, represented as (**C**) a spider chart and (**D**) fold change in absolute number normalized to each isotype control group. Compiled from three independent experiments with a total of 10–16 mice per group. (**E**) Heat map showing the cytokine landscape of draining lymph nodes measured by a multiplex protein assay 3 days after treatment. Values were normalized to the mean value measured for control isotype-treated mice. Compiled from two independent experiments with a total of 4–8 mice per group. (**F–H**) MC38-OVA tumor-bearing mice were treated on day 10 with either anti-PD-1 mAb, anti-PD-1 LALAPG mAb, or isotype control. Quantification of immune cells in tumor-draining lymph nodes by flow cytometry, represented as a spider chart (**F**) and fold changes (**G**) in absolute number compared with the isotype control group. Compiled from three independent experiments with a total of 10–13 mice per group. (**H**) Heat map showing the cytokine landscape of the draining lymph nodes, 3 days after anti-PD-1 mAb treatment. Values were normalized to the mean value measured for control isotype-treated mice. Compiled from two independent experiments with a total of six mice per group. (**I, J**) MC38-OVA tumor-bearing mice were adoptively transferred with naïve CTV-labeled OT-I CD8^+^ T cells on day 10 and treated, or not, with anti-PD-1 mAb. OT-I CD8^+^ T cell proliferation in the draining lymph node was assessed 3 days after treatment. (**I**) Representative histograms showing CTV dilution for OT-I CD8^+^ T cells in the draining lymph node. (**J**) Quantification of OT-I CD8^+^ T cell replication index. Compiled from three independent experiments with a total of 8–10 mice per group. Statistical analyses were performed using Kruskal-Wallis one-way ANOVA (**B, D, G, J**). ns non-significant; *p<0.05; **p<0.01; ***p<0.001. ANOVA, analysis of variance; mAb, monoclonal antibody; WT, wild type.

The intriguing effect of anti-PD-1 mAbs on lymph node swelling could possibly be due to PD-1 binding, FcγR engagement, or both. Indeed, our anti-PD-1 Ab exhibits low-to-moderate binding to FcγR. Interestingly, when mice deficient for the FcRγ-subunit (*fcer1g^−/−^* mice), which is required for the expression of activating IgG receptors FcγRI, FcγRIII, and FcγRIV, were treated with anti-PD-1 mAb, we no longer observed accumulation of immune cells in tumor-draining lymph nodes (figure 3A,B). Similar results were observed when using PD-1 deficient recipient (figure 3C,D). Consistently, the burst in chemokine levels observed after anti-PD-1 treatment was lost in FcRg-deficient and PD-1-deficient hosts (figure 3E, online supplemental figure S2). These results strongly suggest that both FcγR engagement and PD-1 binding are required to boost chemokine levels and promote immune cell recruitment in the lymph node on anti-PD-1 mAb administration. To confirm the importance of FcγR engagement in promoting reactive lymphadenopathy, we used a modified version of the anti-PD-1 mAb clone with abrogated FcγR binding (L_234_A L_235_A P_329_G triple mutation, aka LALAPG). As shown in figure 3F–H, administration of LALAPG anti-PD-1 mAb did not trigger increased lymph node cellularity nor chemokine burst. Similar results were obtained in the E0771 breast cancer tumor model (online supplemental figure S3A,B). Together, these results established that FcγR engagement is critical for immune cell recruitment in the draining-lymph node after anti-PD-1 treatment. Of note, treatment with an anti-Tim3 mAb of the same isotype also promoted robust immune cell mobilization and chemokine production in the lymph node, indicating that the underlying mechanism is not specific to PD-1 checkpoint inhibition (online supplemental figure S3C,D).

We next tested whether the impact of anti-PD-1 mAb on CD8^+^ T cell proliferation was also FcRγ-dependent. We noted that IL-4 levels were similarly increased in both WT and FcRγ-deficient mice treated with anti-PD-1 mAb (figure 3E) and were also maintained in WT mice treated with LALAPG anti-PD-1 mAb (figure 3H). Consistently, the boost in CD8^+^ T cell proliferation was conserved in FcRg-deficient recipients or when using anti-PD-1 LALAPG variant mAb (figure 3I,J, online supplemental figure S2B,C). These observations establish the existence of two distinct and independent mechanisms of anti-PD-1 mAb in the lymph node driving T cell proliferation and immune cell recruitment.

To identify which Fc receptor drives the beneficial chemokine burst, we fluorescently labeled anti-PD-1 WT and LALAPG variant mAb prior to intravenous administration (online supplemental figure S2D). To discriminate between PD-1-specific and PD-1 non-specific binding, lymph node cells were stained ex vivo with a different anti-PD-1 mAb clone that did not interfere with the injected mAb linking in vivo labeling with PD-1 surface expression levels.^19^ We confirmed that non-PD-1-specific binding was predominantly observed with the anti-PD-1 WT mAb compared with the anti-PD-1 LALAPG mAb and identified antibody-specific binding to FcgRIII^+^ cells, consistent with previous reports^20^ (online supplemental figure S2E,F). Among the anti-PD-1 AF594^+^ FcγRIII^+^ cells, we identified DCs, macrophages, and monocytes as potent chemokine producers (online supplemental figure S2G). Together, these results highlight an anti-PD-1 mAb-FcγRIII cross-linking axis responsible for promoting chemokine-induced immune cell recruitment.

### FcγR engagement by human IgG4-anti-PD-1 mAb promotes chemokine production and immune cell recruitment in the lymph node

Most clinically available anti-PD-1 mAb are of the human IgG4 isotype that displays low binding to human FcγR in order to limit phagocytosis of PD-1^+^ T cells by macrophages.^21^ In our preclinical setting, we used an anti-PD-1 mAb clone (RPM1-14) of the rat IgG2a isotype which is known to also display weak binding to activating mouse FcgRs.^22^ Since our results suggested that FcgR-expressing cells may interact with anti-PD-1 mAb-coated T cells to initiate reactive lymphadenopathy, it was important to test whether the impact of FcγR engagement identified in our mouse model also pertains to anti-PD-1 mAbs of the human IgG4 isotype.

**Figure 4:**
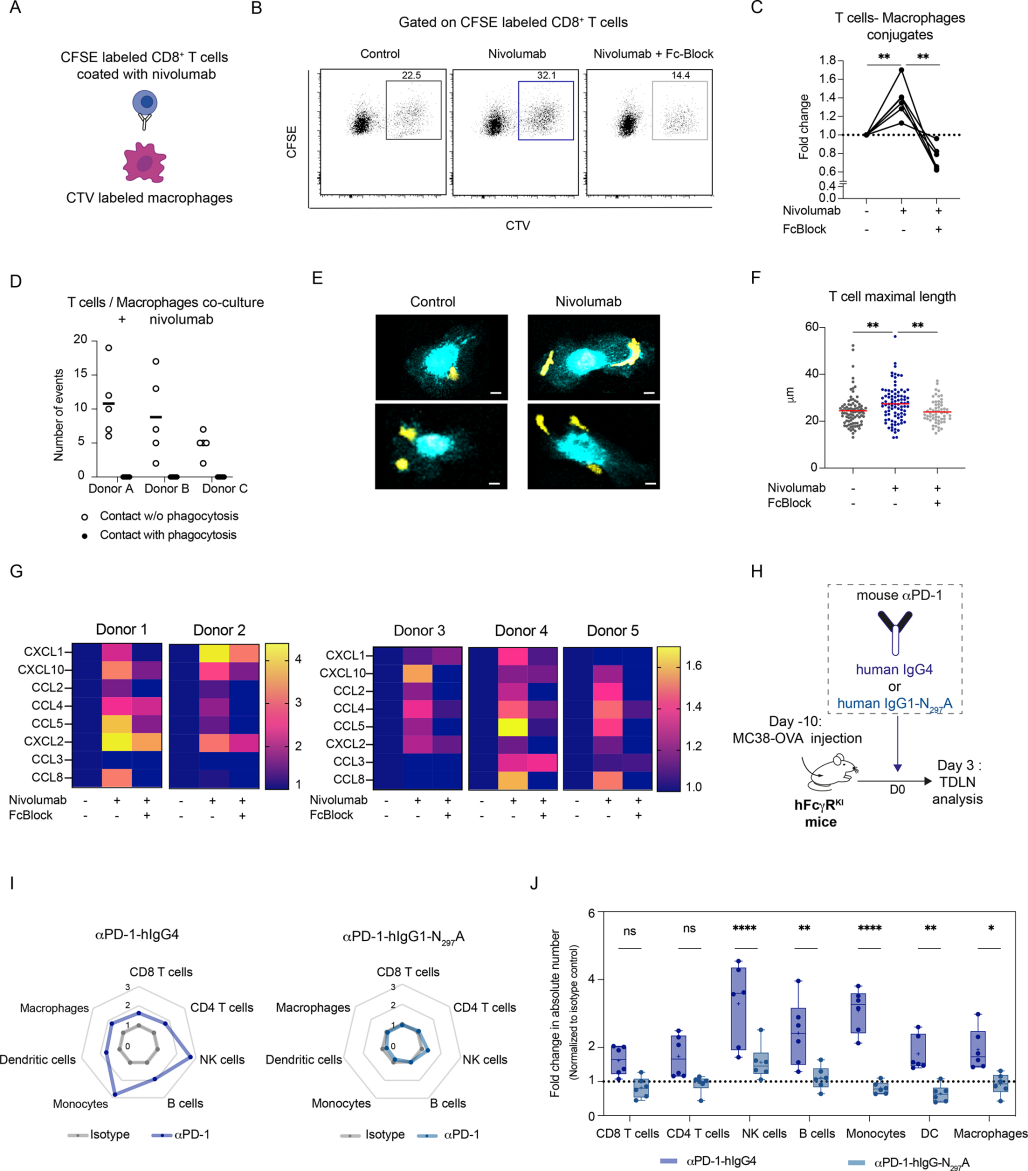
FcgR engagement by human IgG4-anti-PD-1 Ab promotes chemokine production and immune cell recruitment in tumor-draining lymph nodes (TDLNs). (**A–C**) Cell conjugation was assessed on co-culture of cell trace CTV-labeled human macrophages with activated cell trace CFSE-labeled human CD8^+^ T cells in the presence of nivolumab. (**A**) Experimental set-up. (**B**) Representative FACS plots showing that macrophage-T cell conjugation is facilitated by nivolumab in a FcgR-dependent manner. Data are gated on CFSE^+^ cells. Representative of six independent experiments. (**C**) Quantification of macrophage-T cell conjugates expressed as fold changes relative to the isotype control condition. Compiled from six independent experiments. (**D–F**) Live imaging of human T cells (yellow) interacting with macrophages (cyan) in the presence of nivolumab. (**D**) Lack of phagocytosis during macrophage contacts with nivolumab-coated T cells. Each dot represents one time-lapse movie for the indicated donor. (**E, F**) Elongation was defined as the longest T cell length measured during T cell-macrophage contacts. (**E**) Representative images of CD8^+^ T cells during contact with macrophages. (**F**) Quantification of CD8^+^ T cell elongation in the presence or absence of nivolumab and human Fc blocking reagent (FcBlock). (**G**) Heat map showing the cytokine landscape in the supernatant of macrophage-T cell co-cultures in the presence or absence of nivolumab and human Fc blocking reagent. (**H–J**) FcgR-humanized mice (FcγR^KI^ mice) bearing MC38-OVA tumors were treated on day 10 with chimeric anti-PD-1-hIgG4, chimeric anti-PD-1-hIgG1-N_297_A, or with hIgG4 isotype control. (**H**) Experimental set-up. Quantification of immune cells in the TDLN of hFcgR^KI^ mice, represented as a spider chart (**I**) and fold change (**J**) in absolute number normalized to the isotype control group. Compiled from two independent experiments with a total of six mice per group. Statistical analyses were performed using Kruskal-Wallis one-way ANOVA (**F, J**) or nested one-way ANOVA (**C**). ns non-significant; *p<0.05; **p<0.01; ****p<0.0005. ANOVA, analysis of variance; CTV, CellTrace Violet; DC, dendritic cell.

After confirming that human macrophages displayed FcgR-dependent binding of a fluorescently labeled version of nivolumab, an IgG4 therapeutic anti-PD-1 mAb (online supplemental figure S4), we first tested whether human FcγR-expressing cells such as macrophages could engage and respond to IgG4 anti-PD-1 mAb-coated human T cells (figure 4A). We found that the presence of nivolumab on human activated T cells promoted interactions with FcγR-expressing cells as measured in a cell conjugation assay (figure 4A–C). As shown in figure 4D and online supplemental materials 1, live imaging indicated that the presence of anti-PD-1 mAbs at the surface of T cells did not lead to T cell phagocytosis during contact with macrophages (n=121 contacts analyzed, representing a cumulated time of imaging of 122 hours). However, we noted that in the presence of nivolumab, T cells adopted a more elongated morphology on interaction with macrophages (figure 4E,F, online supplemental movie 1). This phenomenon was found to be abrogated in the presence of an Fc blocking reagent, further suggesting FcγR engagement during interactions with nivolumab-coated T cells (figure 4E,F). To test the functional impact of myeloid cell FcγR engagement by nivolumab, we measured chemokine production on co-culture of human macrophages and human activated T cells, in the presence or absence of nivolumab and Fc block. As shown in figure 4G and online supplemental table 1, the presence of nivolumab enhanced the production of multiple chemokines including CCL2, CCL4, CCL5, CCL8, CXCL2, and CXCL10 in the five distinct donors tested. Importantly, chemokine production was strongly reduced in the presence of Fc block. Overall, our results suggest that FcγR engagement by nivolumab initiates a burst of chemokine production, resembling that seen in our preclinical model.

Second, we sought to determine how hIgG4 binding to human FcgR could modify the lymph node microenvironment in vivo. To this end, we relied on mice humanized for all classical FcγRs (hFcgR^KI^ mice, expressing human FcγRI, FcγRIIA, FcγRIIB, FcγRIIIA, and FcγRIIIB),^23 24^ and treated hFcγR^KI^ tumor-bearing mice with a human IgG4 chimeric variant of the anti-PD-1 mAb (clone RPM1-14) (figure 4H). As a control, we also used an effector-less human IgG1 N_297_A variant of this mAb that is unable to bind human FcgRs^25^ (figure 4H). We observed that anti-PD-1-hIgG4 induced robust lymph node swelling with the accumulation of numerous immune cell populations (including T cells). This was not the case for mice treated with anti-PD-1-hIgG1-N_297_A, which cannot bind hFcgRs (figure 4I,J). Altogether, these results support the idea that human IgG4 anti-PD-1 mAbs interact with human FcγRs to promote chemokine production and immune cell mobilization in the lymph node, extending our original observations to human settings.

### Immune cell recruitment in lymph nodes during anti-PD-1 therapy is type I IFN dependent

**Figure 5:**
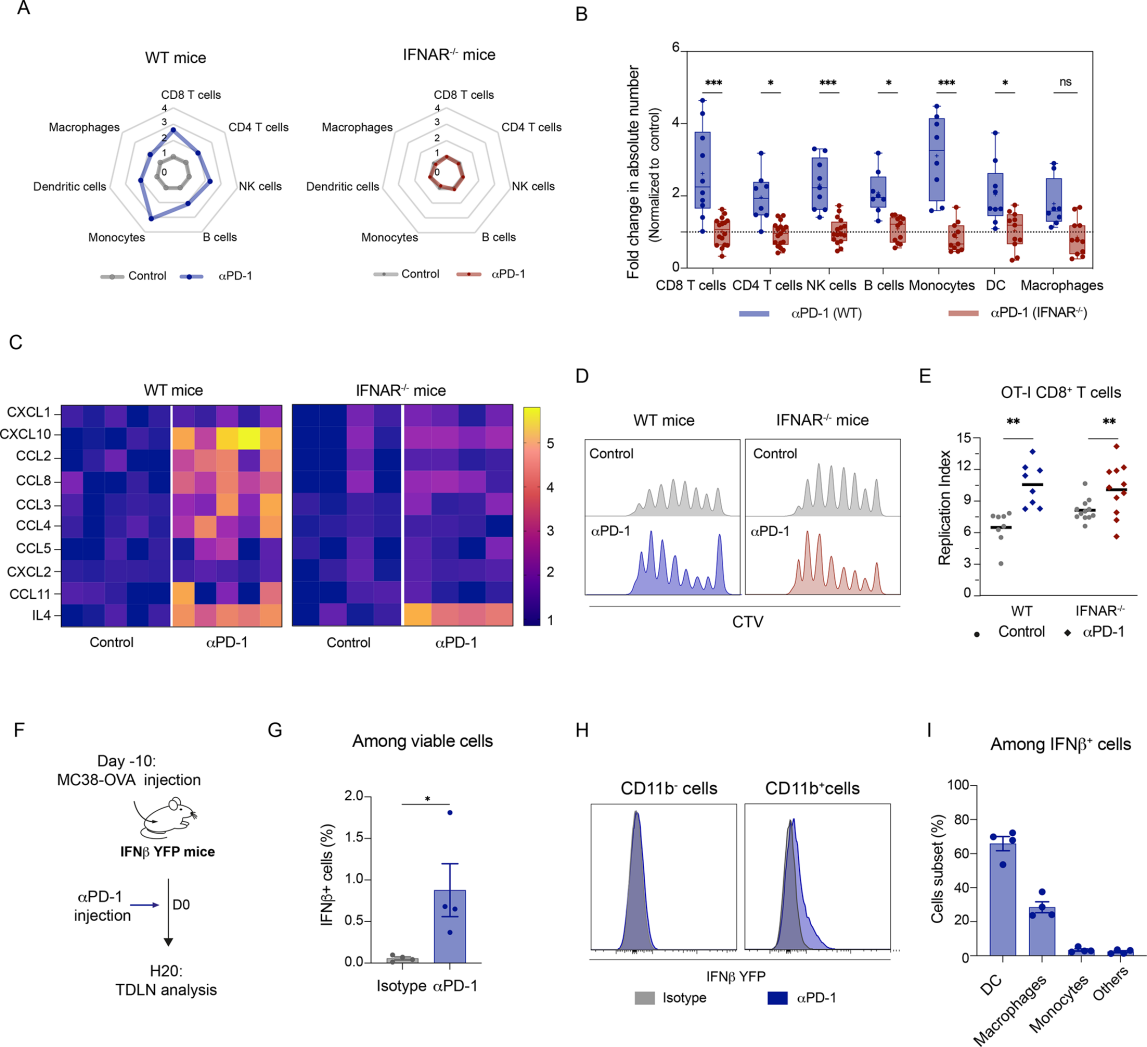
Immune cell recruitment in lymph nodes during anti-PD-1 therapy depends on type I IFN signaling. (**A–C**) MC38-OVA tumor-bearing WT or IFNAR^−/−^ mice were treated on day 10 with anti-PD-1 mAb or with isotype control. Quantification of immune cells in the tumor-draining lymph node (TDLN) of WT or IFNAR^−/−^ mice, represented as (**A**) spider charts and (**B**) fold changes in absolute number compared with the isotype control-treated group. Compiled from three to four independent experiments with a total of 10–18 mice per group. (**C**) Heat map showing the cytokine landscape of the draining lymph nodes, 3 days after anti-PD-1 mAb treatment. Values were normalized to the mean value measured for control isotype-treated mice. Compiled from two independent experiments with a total of 4–5 mice per group. (**D, E**) MC38-OVA tumor-bearing WT or IFNAR^-/-^ mice were adoptively transferred with naïve CTV-labeled OT-I CD8^+^ T cells on day 10 and treated or not with anti-PD-1 mAb. OT-I CD8^+^ T cell proliferation was assessed on day 3 in the draining lymph node. (**D**) Representative histograms showing CTV dilution in OT-I CD8^+^ T cells. (**E**) Quantification of OT-I CD8^+^ T cell replication index. Compiled from three independent experiments with a total of 8–11 mice per group. (**F–I**) MC38-OVA tumor-bearing IFNb-YFP mice were treated on day 10 with anti-PD-1 mAb or with isotype control. (**F**) Experimental set-up. (**G**) Quantification of IFNβ^+^ cells among viable cells. (**H**) Representative FACS plots showing the contribution of CD11b^+^ myeloid cells to IFNβ production. (**I**) Proportion of each IFNβ^+^ CD11b^+^ myeloid cell subset (DC CD11c+MHCII+; Macrophages F4/80^+^CD169^+^, F4/80^+^CD169^-^, F4/80^-^CD169^-^; Monocytes Ly6C^+^; neutrophils Ly6G^+^). Obtained from one experiment with four mice per group. Statistical analyses were performed using Kruskal-Wallis one-way ANOVA (**B, E**). ns, non-significant; *p<0.05; **p<0.01, ***p<0.001. ANOVA, analysis of variance; CTV, CellTrace Violet; DC, dendritic cell; mAb, monoclonal antibody; WT, wild type.

Type I IFN signaling has been shown to be important for immune cell recruitment in the lymph node during a viral challenge.^26^ We therefore assessed the contribution of IFNAR signaling to the peripheral mechanisms of anti-PD-1 therapy. Anti-PD-1 treatment in tumor-bearing IFNAR^−/−^ recipients did not result in changes in lymph node cellularity with no evidence of immune cell recruitment (figure 5A,B). Consistent with a type I IFN response in the tumor-draining lymph node on PD-1 blockade, we noted widespread phosphorylation of IRF3, a key step for the induction of type I IFN (online supplemental figure S5A,B). In good agreement with these observations, lymph node chemokine levels were not upregulated by anti-PD-1 mAb treatment in these mice (figure 5C, online supplemental figure S5C). By contrast, IL-4 production as well as the proliferative boost in antigen-specific CD8^+^ T cells during anti-PD-1 therapy were similar in anti-PD-1-treated IFNAR^−/−^ and WT mice (figure 5C–E, online supplemental figure S4). A similar defect during anti-PD-1 therapy was observed in the E0771 breast cancer model treated with an anti-IFNAR antibody (online supplemental figure S5D,E). These results suggest that during anti-PD-1 therapy type I IFN signaling is required for immune cell mobilization in lymph node but is dispensable for boosting T cell proliferation. Finally, we sought to further characterize the cellular sources involved in the type I IFN response. To this end, we used IFNβ-YFP reporter mice, which enabled us to attribute IFNβ production following anti-PD-1 treatment primarily to CD45^+^CD11b^+^ cells (figure 5F–H). Notably, we identified DCs as the main IFNβ-producing population, followed by macrophages and monocytes. These findings are consistent with our previous identification of these FcγRIII^+^ cells as key contributors. To determine whether type I IFN sensing within the immune or stromal compartment was required to trigger lymphadenopathy, we generated bone marrow chimeras using either CD45.1^+^ wild-type or IFNAR-deficient bone marrow cells. In this setting, immune cells appeared to be the critical compartment, as the recruitment mechanism was abolished when IFNARKO bone marrow cells were transferred into WT CD45.1 hosts. Collectively, these findings establish a model in which anti-PD-1-mediated FcγRIII engagement in myeloid cells induces type I IFN production that must be sensed by hematopoietic cells to drive the chemokine burst and subsequent immune cell recruitment.

### Increased mobilization of peripheral antigen-specific T cells by anti-PD-1 contributes to anti-tumor activity

**Figure 6:**
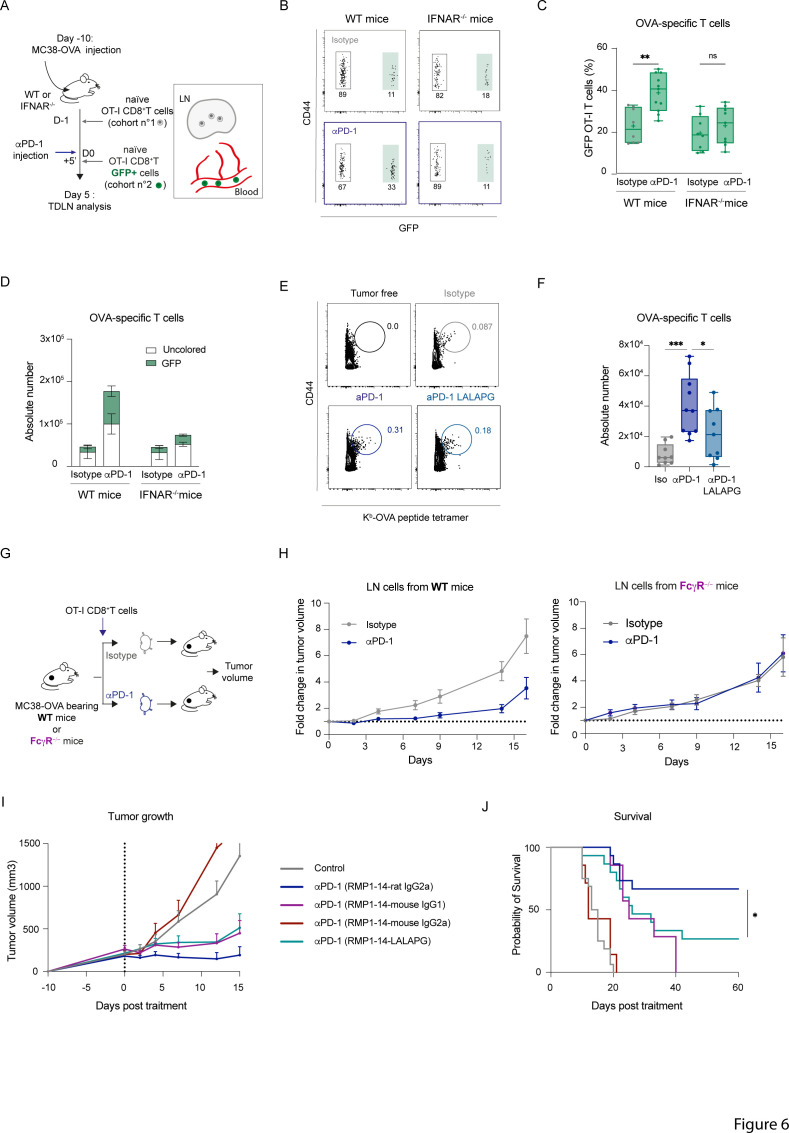
Enhanced mobilization of peripheral antigen-specific T cells by anti-PD-1 therapy contributes to its anti-tumor efficacy. (**A–D**) MC38-OVA tumor-bearing WT or IFNAR^−/−^ mice were adoptively transferred on day 9 with a first cohort of naïve unlabeled OT-I CD8^+^ T cells. On day 10, mice were adoptively transferred with a second cohort of naïve GFP-labeled OT-I CD8^+^ T cells and treated, or not, with anti-PD-1 mAb. (**A**) Experimental set-up. (**B**) Representative FACS plots showing the contribution of GFP^−^ and GFP^+^ OVA-specific CD8^+^ T cells (H2-K^b^-OVAp tetramers^+^) within CD8^+^ T cells. (**C**) Contribution of GFP^+^ OT-I CD8^+^ T within OVA-specific T cells. (**D**) Absolute numbers of GFP^−^ and GFP^+^ OVA-specific T cells in tumor-draining lymph nodes (TDLNs). Compiled from two independent experiments with 9–11 mice per group. (**E–F**) MC38-OVA tumor-bearing mice were treated on day 10 with anti-PD-1 mAb, with anti-PD-1, anti-PD-1 LALAPG, or with an isotype control. Representative FACS plots (**E**) and quantification (**F**) showing H2-K^b^-OVAp tetramers^+^ among CD8^+^ T cells 5 days after treatment. Compiled from two independent experiments with 9–10 mice per group. (**G–H**) MC38-OVA tumor-bearing WT or FcR^γ−/−^ mice were adoptively transferred with naïve OT-I CD8^+^ T cells and treated, or not, with anti-PD-1 mAb. 3 days later, cells from the TDLNs of each animal were isolated and transferred into a new tumor-bearing mouse. (**G**) Experimental set-up. (**H**) Evolution of tumor volume. Compiled from two independent experiments with 8–10 mice per group. (**I, J**) MC38-OVA tumor-bearing mice were treated on day 10 with anti-PD-1 mAb (rat IgG2a, mouse IgG2a, mouse IgG1), anti-PD-1 LALAPG, or with an isotype control. (**I**) Tumor growth and (**J**) Survival curve. Compiled from three independent experiments with 7–17 mice per group. Statistical analyses were performed using Kruskal-Wallis one-way ANOVA (**B, F**) or using a log-rank test for mice survival (**J**). ns non-significant; *p<0.05; **p<0.01; ***p<0.001. ANOVA, analysis of variance; DC, dendritic cell; mAb, monoclonal antibody; WT, wild type.

We hypothesized that an overall increase in immune cell infiltration induced by anti-PD-1 mAbs would result in the recruitment of additional T cells to the draining lymph node for priming and activation. First, we designed an experiment aimed to evaluate how circulating T cells (that were not initially present in the lymph node) contribute to the antigen-specific T cell response. A first cohort of naïve OT-I T cells was injected 24 hours prior to anti-PD-1 mAbs so that T cells could redistribute and home to lymphoid organs (with a large fraction relocating in lymph nodes) (figure 6A). The second cohort of naïve OT-I T cells expressed GFP and was injected concomitantly with the anti-PD-1 mAbs so that these T cells were primarily in the circulation and excluded from lymph node at the time of treatment (figure 6A). On day 5 after anti-PD-1 mAbs treatment, we analyzed the specific contribution of GFP^+^ T cells in the bulk of the antigen-specific T cell pool to evaluate the mobilization of T cells that were not initially present in the lymph node. In line with our hypothesis, anti-PD-1 mAb treatment substantially increased the proportion of GFP^+^ T cells within the total response (figure 6B–D). By contrast, when the experiment was conducted in IFNAR^−/−^ recipients, anti-PD-1 treatment did not modify the contribution of GFP^+^ T cells confirming the essential role of type I IFN in mobilizing circulating CD8^+^ T cells to the tumor-draining lymph nodes (figure 6B–D).

Second, we assess how FcγR engagement during anti-PD-1 therapy impacts the emergence of tumor-specific T cells in the lymph node using either WT or LALAPG anti-PD-1 mAbs. Relying on H2-K^b^-OVAp tetramer staining on day 5 after treatment, we observed that anti-PD-1 mAbs strongly increased the absolute number of antigen-specific T cells, whereas LALAPG anti-PD-1 mAbs had a ~2-fold less effect (figure 6E,F). Thus, FcgR engagement during anti-PD-1 mAb treatment contributes to increase the magnitude of antigen-specific T cells generated in the tumor-draining lymph node.

Third, we tested whether the mobilization of lymph node T cells during anti-PD-1 mAb therapy contributed to the overall anti-tumor activity. To this end, OT-I T cells were adoptively transferred in WT and FcRγ^−/−^ tumor-bearing mice that were treated or not with anti-PD-1 mAbs. We collected tumor-draining lymph nodes, and, for each condition, the equivalent of the cell content of one lymph node was re-infused into a tumor-bearing animal (figure 6G). As shown in figure 6H, lymph node cells from WT mice treated with anti-PD-1 mAbs reduced tumor growth and increased survival of the recipient mouse. This was not the case with lymph node cells from untreated mice or with lymph node cells originating from anti-PD-1-treated FcRγ^−/−^ animals (figure 6H). These results support the idea that the FcgR-dependent mechanism driving immune cell recruitment in the lymph node improves the overall anti-tumor activity of anti-PD-1 mAb therapy. Finally, to investigate how strength of FcγR engagement influences the overall anti-PD-1 efficacy, we compared the survival of tumor-bearing mice treated with four Fc isotypes of the same RMP1-14 clone (Rat IgG2a, Mouse IgG1, Mouse IgG2a, LALAPG), which possess distinct FcγR affinity profiles^22^ (figure 6I,J). Notably, anti-PD-1 rat IgG2a, which exhibits low-to-moderate FcgR binding, significantly improved survival compared with the Fc-null LALAPG variant or the mouse IgG1 isotype. By contrast, the mouse IgG2a isotype, known for its cell-depleting activity, showed no survival benefit, behaving similarly to the control (figure 6I,J). These results highlight that low to moderate FcγR engagement, but not maximal or null binding, optimally enhances the anti-tumor efficacy of anti-PD-1 antibodies.

### FcγR engagement during anti-PD-1 mAb administration contributes to increased T cell responses in a vaccine setting

**Figure 7:**
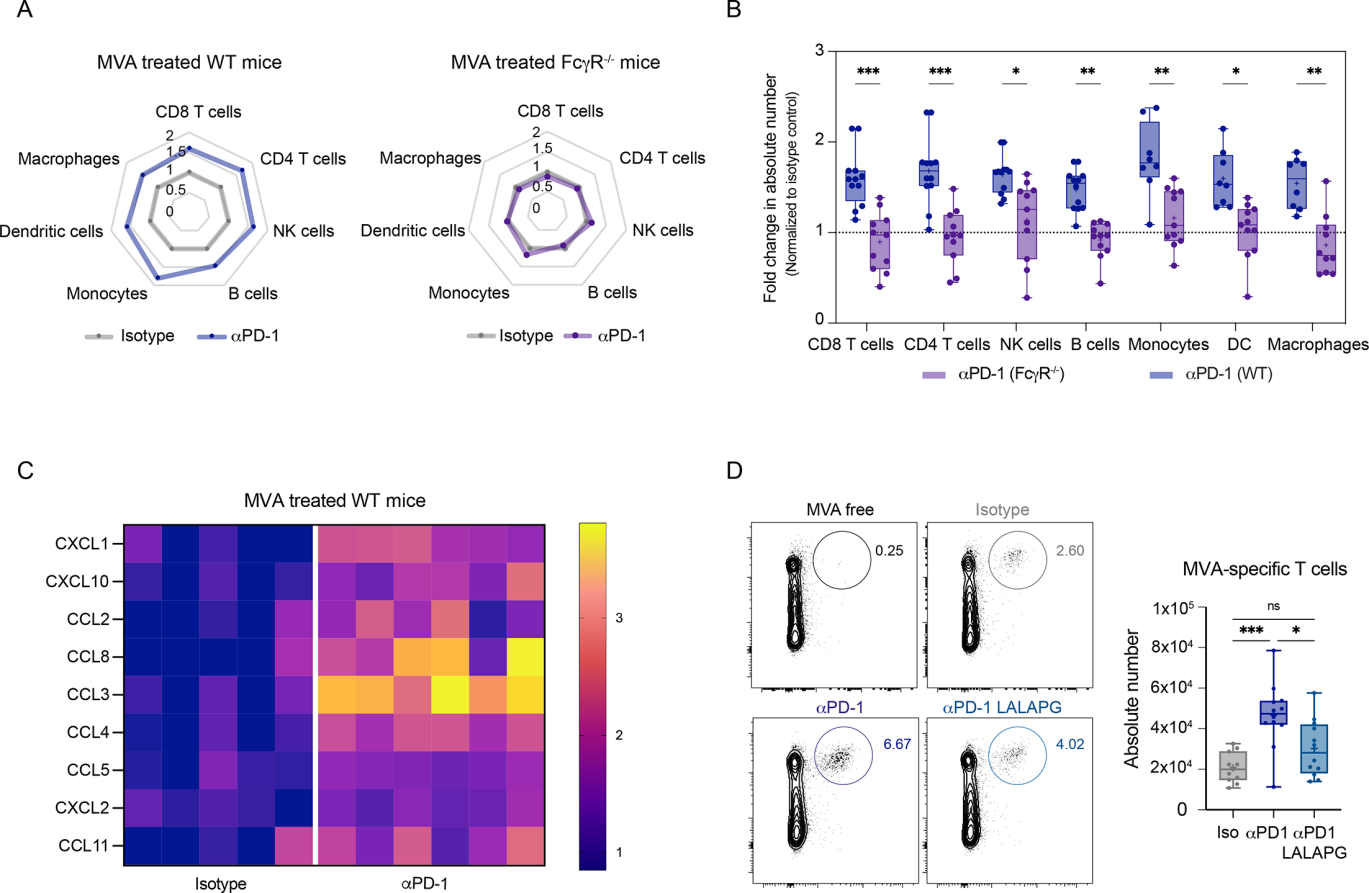
FcgR engagement during anti-PD-1 antibody treatment boosts T cell responses in a vaccination context. (**A–D**) C57BL/6 mice were injected into the footpad with 2.10^6^ p.f.u. of Modified Virus Ankara (MVA)-HIV-B. After 3 days, mice were treated i.v. with anti-PD-1 or a control isotype. The draining lymph nodes were analyzed on day 6. Quantification of immune cells by flow cytometry in tumor-draining lymph nodes, represented as (**A**) a spider chart and (**B**) fold change in absolute number normalized to the isotype control group. Compiled from three independent experiments with a total of 11–12 mice per group. (**C**) Heat map showing the cytokine landscape of the draining lymph nodes, 3 days after anti-PD-1 mAb treatment. Each value was normalized to the mean of value of control samples. Compiled from two independent experiments with a total of 5–6 mice per group. (**D**) Quantification of H2-K^b^-B8R tetramers^+^ CD8^+^ T cells. Compiled from two independent experiments with 12 mice per group. Statistical analyses were performed using Kruskal–Wallis one-way ANOVA (**B, D**). ns, non-significant; *p<0.05; **p<0.01; ***p<0.001. ANOVA, analysis of variance; DC, dendritic cell; i.v., intravenous; mAb, monoclonal antibody; WT, wild type.

To test whether FcγR engagement by anti-PD-1 mAbs is a general mechanism improving the lymph node T cell responses, we relied on a vaccine setting. Mice were injected with an Modified Virus Ankara (MVA)-based vaccine and treated or not with anti-PD-1 mAbs. As observed in the tumor setting, anti-PD-1 mAb administration during vaccination induced reactive lymphadenopathy associated with a burst of chemokine production (figure 7A–C). This immune cell mobilization in the lymph node was abolished in vaccinated and anti-PD-1 mAb-treated FcRγ-deficient mice (figure 7A,B). Moreover, the magnitude of MVA-specific CD8^+^ T cells generated by the vaccine in combination with anti-PD-1 mAbs was reduced with the effector-less variant (LALAPG) of anti-PD-1 (figure 7D). Thus, FcγR engagement following anti-PD-1 mAb administration contributes to increase lymph node CD8^+^ T cell responses in both tumorous and vaccine settings.

## Discussion

Here, we uncovered a new mechanism mediated by anti-PD-1 mAbs in lymph nodes. We show that FcγR engagement and type I IFN during anti-PD-1 therapy promote immune cell recruitment in the tumor-draining lymph node, including the mobilization of circulating T cells. Importantly, the enhanced pool of available T cells in the tumor-draining lymph node contributes to improved anti-tumor immune responses. A similar mechanism was also observed in a vaccine setting, suggesting that this is a general feature of anti-PD-1 mAb activity in draining lymph nodes.

Previous studies have documented the importance of the tumor-draining lymph node during anti-PD-1 mAb therapy.^1016^,^18^ The present work identifies a novel mechanism that contributes to these observations whereby FcgR engagement by anti-PD-1 mAb promotes the mobilization of circulating T cells in the tumor-draining lymph node, thus enhancing anti-tumor T cell responses. Notably, we have previously shown that binding of anti-PD-1 mAb on Tfh cells stimulates IL-4 production, boosting the proliferation of tumor-specific T cells in lymph nodes.^17^ Remarkably, these two mechanisms of anti-PD-1 mAbs, promoting rapid T cell recruitment and subsequent expansion, were largely independent. Indeed, FcγR engagement and type I IFN signaling were essential for T cell recruitment but dispensable for proliferation, whereas IL-4 production was specifically required to boost T cell expansion. However, these two mechanisms appear largely complementary to increase the pool of responding T cells. Overall, a dual mechanism underlies anti-PD-1 mAb activity in lymph nodes, acting mostly in a cell-extrinsic manner.

One key finding was that FcgR engagement by anti-PD-1 mAb in the lymph node triggered reactive lymphadenopathy. Interestingly, both PD-1 binding and FcgR engagement were required for lymph node swelling, suggesting that anti-PD-1-coated cells are important to crosslink FcgRs on myeloid cells and promote chemokine release. The massive influx of immune cells in the lymph node was critically dependent on type I IFN signaling, reminiscent of observations made in infectious settings.^26^ Indeed, engagement of Fc receptors by myeloid cells triggered the production of type I IFN, which emerged as a central driver of the recruitment process. This IFN was sensed by hematopoietic cells, inducing chemokine production and ultimately promoting the recruitment of tumor-specific T cells. This phenomenon likely contributes to the strong modulation of the lymph node microenvironment observed in anti-PD-1 mAb-treated patients^27^ and is consistent with previous work showing that FcγR engagement by anti-CTLA-4 mAbs can reprogram the tumor microenvironment through type I IFN signaling and macrophage polarization.^28^

The capacity of immune checkpoint blockers to engage FcgRs has a profound impact on their therapeutic activity due to their capacity to trigger effector functions such as ADCC, ADP, or cytokine production. For some target molecules, including CTLA-4, VISTA, or TIGIT, FcgR engagement has been shown to improve overall anti-tumor activity in preclinical models.^21^ For example, FcgR binding of anti-CTLA-4 mAbs promotes the depletion of regulatory T cells and reprograms the tumor microenvironment through type I IFN signaling and macrophage polarization.^28^,^30^ In contrast, strong FcgR binding can be deleterious for target molecules expressed on effector T cells, such as PD-1, due to the potential elimination of tumor-reactive T cells^20 31 32^ or due to FcγR-dependent capture of anti-PD-1 Abs.^33^ The differing outcomes of Fc engagement in tumors versus lymph nodes may reflect receptor-specific effects and microenvironmental context. In tumors, deleterious effects have been observed where anti-PD-1 is captured by macrophages, limiting its availability on T cells. Notably, Arlauckas and Pittet demonstrated this negative effect using an FcγRII/III-blocking antibody, but the exact receptor responsible was not identified^33^. Given their results, it is plausible that FcγRIIB contributes to this deleterious effect in the tumor. By contrast, in tumor-draining lymph nodes, we show that FcγRIII^+^ myeloid cells mediate the beneficial effects of anti-PD-1 through cross-linking, triggering type I IFN production, chemokine release, and recruitment of immune cells. Together, these observations suggest that the same antibody can have opposite outcomes depending on which Fc receptor is engaged and the tissue context, emphasizing the importance of fine-tuning Fc–FcγR interactions to maximize therapeutic efficacy while minimizing deleterious effects.

Most human anti-PD-1 mAbs are designed to minimize or abrogate FcγR engagement. For example, nivolumab and pembrolizumab are of the IgG4 isotype with lower, but not nonexistent, reactivity toward FcgRs compared with IgG1.^22 34^ Indeed, IgG4 interacts with three activating IgG receptors ie, with high affinity with human FcγRI (CD64) and low affinity with FcγRIIA (CD32A) and FcγRIIIA (CD16A), raising the question of a potential contribution for FcgR binding on therapeutic activity. Our results support the idea that myeloid FcgR engagement in trans by hIgG4 anti-PD-1 mAbs opsonizing T cells exerts a beneficial effect that contributes to broadening CD8^+^ T cell responses in tumor-draining lymph nodes. This effect was indeed absent with antibodies designed with abrogated effector functions (Fc null). Moreover, we showed that IgG4 anti-PD-1 mAb linked PD-1^+^ T cells with macrophages, promoting chemokine production without inducing phagocytosis, and triggered reactive lymphadenopathy in mice with humanized FcγRs in vivo. Overall, our results identify an unexpected beneficial effect mediated by hIgG4 anti-PD-1 mAbs in lymph nodes. When integrated with prior studies that have shown deleterious effects of strong FcγR binding,^20 31 32^ our data suggest that low to moderate FcγR binding may represent the optimal balance for anti-PD-1 therapy, avoiding depletion of PD-1^+^ T cells while enhancing lymph node-mediated recruitment.

In sum, we identify a previously unrecognized mechanism whereby anti-PD-1 antibodies broaden tumor-specific T cell responses by mobilizing circulating T cell clones within draining lymph nodes. Uncovering the diversity of cell-intrinsic and cell-extrinsic mechanisms mediated by immune checkpoint blockers in distinct anatomical sites remains critical to optimize antibody design or to identify and leverage key mediators triggered by these therapies.

## Materials and methods

### Mice

C57BL/6J mice, aged 6–8 weeks, were purchased from ENVIGO and housed in the animal care facility at Institut Pasteur. GFP, GFP-OT-I-Rag1^−/−^, FcRg^−/−^, PD-1^−/−^, IFNAR,^−/−^ and hFcγR^KI^ (Trinity mice designed by intercross of VG1505 and VG1543 mice generated by Regeneron Pharmaceuticals, Tarrytown, NY)^23 24^ (B6 background) were bred on-site at our facility. IFNβ-YFP reporter mice were kindly provided by the laboratory of Pierre Guermonprez (Institut Pasteur).

### Cell lines

MC38o tumorcells oexpressing valbumine antigen (MC38-OVA) ^17^ were cultured in DMEM+Glutamax medium (Gibco) supplemented with 10% heat-inactivated fetal bovine serum, 50 U/mL penicillin, 50 µg/mL streptomycin, 1 mM sodium pyruvate, 10 mM Hepes, 1 mM non-essential amino acids, and 50 µM gentamicin. E0771 tumor cells were cultured in RPMI+Glutamax medium (Gibco) supplemented with 10% heat-inactivated fetal bovine serum, 50 U/mL penicillin, 50 µg/mL streptomycin, 1 mM sodium pyruvate, 10 mM Hepes. The cells were routinely screened for mycoplasma contamination.

### Human T cell-macrophage co-culture

Blood samples from healthy donors were obtained from the Etablissement Français du Sang (EFS, Paris). Human peripheral blood mononuclear cells from healthy donors were carefully isolated by density gradient centrifugation using Lymphocyte Separation Medium (Corning) following the manufacturer’s protocol. Cells were plated in petri dishes and maintained in 50 ng/mL of human macrophage colonystimulatingfactor(M-CSF) (Peprotech) in complete RPMI. CD8^+^ T cells were isolated from the same donors using the Human CD8^+^ T Cell Isolation Kit (Miltenyi Biotec) and activated using Dynabeads Human T-activator CD3/CD28 (Gibco) for them to express PD-1. Macrophages were detached by using cold phosphate buffered saline buffer (PBS), 10% heat-inactivated FCS, 5 mM EDTA, and labeled with 0.1 µM CellTrace Violet Cell Proliferation Kit (Invitrogen). When indicated, macrophages were pre-incubated with Trustain Fc blocker (Biolegend; 1:25). Subsequently, activated human CD8^+^ T cells were stained with 5 mM CellTrace CFSE Cell Proliferation Kit (Invitrogen), incubated with 30 mg/mL of Nivolumab biosimilar (BioxCell), rinsed, and finally added to macrophages at a 1:1 ratio. Cell conjugates were analyzed 1 hour later by flow cytometry.

### Tumor implantation and monitoring

Mice received a subcutaneous injection of MC38-OVA or E0771 tumor cells (0.5×10⁶ tumor cells in 200 µL of PBS) in the right flank. Tumor volume was monitored three times per week, and mice were euthanized on reaching humane endpoints. Monitoring was conducted in a blinded fashion using randomized number assignment.

### Treatments

10 days after tumor inoculation, when tumors reached a diameter of at least 6 mm, mice were injected intravenously with 250 µg of anti-PD-1 mAb (BioXCell, clone RMP1-14), either in a WT format (rat IgG2a, BioXCell) used for the entire demonstration or (mouse IgG2a and mouse IgG1, BioXcell), or as an effector-less LALAPG mutant of rat-mouse chimeric mouse IgG2a,κ swap variant (BioXCell, clone RMP1-14 CP153), or as a chimeric rat-human S_228_P-stabilized IgG4 swap variant (engineered in house), or as a chimeric rat-human N_297_A-effector-less IgG1 swap variant (engineered in house) or with 250 µg of anti-Tim3 mAb (rat IgG2a, BioXcell clone RMT3-23). In control groups, mice received 250 µg of a rat IgG2a isotype control (BioXCell). For some experiments, mice were injected intravenously with 350 µg of anti-IFNAR mAb (BioXCell, clone MAR1-5A3) 1 day before receiving anti-PD-1 treatment. The number of animals per group and the group design were determined with the Department of Statistics to ensure the use of robust statistical tests while minimizing animal numbers. Group sizes were optimized according to the parameters evaluated, considering tumor growth variability, an estimated 70% tumor take rate, and potential cage effects. For each experiment, mice receiving different treatments were housed together in the same cage.

### Adoptive transfer

Naïve OT-I CD8^+^ T cells were isolated from OT-I RAG1 Rag1^−/−^ or GFP^+^ OT-I RAG1 Rag1^−/−^ mice and injected intravenously (3×10⁶ cells per mouse). For proliferation assays, OT-I CD8^+^ T cells were labeled with Cell Trace Violet Proliferation Dye (Thermo Fisher) following the manufacturer’s protocol. Splenocytes were isolated from UBC-GFP mice and 10×10⁶ cells were injected intravenously. For adoptive transfer of lymph node cells, tumor-draining lymph nodes were isolated from WT or FcRg^−/−^ mice, dissociated ex vivo, and the resulting cell suspension was injected intravenously into a tumor-bearing mouse. The entire cell suspension obtained from a single lymph node was administered to one recipient mouse.

### MVA vaccination

The recombinant MVA-HIV-B, which encodes full-length HIV Gag along with three Pol and two Nef fragments,^35^ was supplied by the Agence Nationale de Recherche sur le Sida. Mice were injected with 2×10⁶ p.f.u. of MVA-HIV-B via footpad injection.

### Fluorescently labeled anti-PD-1 antibody

Anti-PD-1 WT and LALAPG variant mAb were conjugated with the Alexa Fluor 594 Conjugation kit (Fast)—Lightning-Link (Abcam) following the manufacturer’s instructions. Tumor-bearing mice were injected intravenously with 250 µg labeled anti-PD-1 WT or LALAPG variant mAb (clone RMP1-14). In vivo binding of labeled anti-PD-1 Ab was assessed 20 hours later by flow cytometry. A second anti-PD-1 clone (29F.1A12) whose staining is not blocked by prior RMP1-14 binding was used to stain lymph node cells ex vivo.^19^

### Flow cytometry

Single-cell suspensions were prepared from tumor-draining lymph nodes and non-draining lymph nodes by mechanical followed by enzymatic dissociation for 20 min at 37°C with DNase I (100 µg/mL, Roche), collagenase (1 mg/mL, Roche). For standard cell surface staining, the cells were incubated in FACS buffer (2% FBS, 0.2% EDTA in PBS) with anti-CD16/CD32 antibody (1:200, Biolegend), fixable viability dye eF780 (1:1000, Invitrogen), and the following antibodies for 1 hour at 4°C:

CD45.1-FITC (1:100, clone A20), CD45.2-BUV737 (1:100, clone 104), NK1.1-BV650 (1:200, clone PK136), TCRb-BV650 (1:200, clone H57-597), CD4-BUV395 (1:200, clone GK1.5), CD4-BUV805 (1:200, clone GK1.5), CD8a-BUV395 (1:200, clone 53–6.7), IA/IE- BUV496 (1:400, clone 2G9), CD32-PE (1:100, clone AT130-2), and LY6C-BUV563 (1:200, clone HK1.4) were purchased from BD Biosciences. Moreover, CD19-BV650 (1:200, clone 6D5), CD19-Alexa 647 (1:100, clone 6D5), NK1.1-BV711 (1:200, clone PK136), TCRb-PE (1:200, clone H57-597), CD4-APC (1:200, clone GK1.5), CD4-Alexa488 (1:200, clone GK1.5), CD4-PE (1:200, clone GK1.5), CD8a-BV786 (1:200, clone 53–6.7), CD8-FITC (1:200, clone 5H10-1), CD11b-PerCPCy5.5 (1:200, clone M1/70), CD11b-BUV737 (1:200, clone M1/70), CD11c-PeCy7 (1:200, clone NA418), CD11c-BV786 (1:200, clone NA418), CD44-BV605 (1:100, clone IM7), CD45.2-BV605 (1:200, clone 104), IA/IE-BV510 (1:400, clone 2G9), Ly6C-BV605 (1:200, clone HK1.4), Ly6G-AF488 (1:200, clone 1A8), Ly6G-BV510 (1:200, clone 1A8), CD169-BV605 (1:200, clone 3D6.112), CD64-APC (1:100, clone X54-5/7.1), CD16.2-BV786 (1:100, clone 9E9), and PD-1-PeCy7 (1:100, clone 29F.1A12), were purchased from Biolegend. FcγRIII-AF488 (1:100, clone 275003) was purchased from R&D Systems.

H2-K^b^-OVA_257-264_ and H2-K^b^-TSYKFESV MHC:peptide tetramers were generously provided by the National Institutes of Health Tetramer Core Facility. Tetramer staining was conducted first, followed by staining with the antibody cocktail. Counting beads (Invitrogen) were added to the samples to determine absolute cell numbers. Data were collected using a Fortessa or Symphony flow cytometer (BD Biosciences) and analyzed using FlowJo software V.10.8.1 (BD Biosciences).

### Multiplex assays for chemokine quantification

For mice chemokine quantification, 1×10⁷ cells from the tumor-draining lymph node were resuspended in 400 µL of Lysis Buffer (Invitrogen) containing Protease Cocktail Inhibitor (1:100, Thermo Fisher). Pierce Universal Nuclease for Cell Lysis (Thermo Fisher) was added to each sample (1 µL per sample). After a 10 min incubation on ice, samples were briefly sonicated (30 s at 25 KHz). The supernatant was collected by ultracentrifugation at 14 000 g for 10 min in a cold microfuge. Multiplex assays were conducted using the 21-Plex ProcartPlex Panel (Invitrogen) according to the manufacturer’s instructions. Data analysis was conducted using a Bio-Plex 200 system with Bio-Plex Manager software (Bio-Rad).

For human chemokine quantification, supernatants from the co-culture of macrophages and T cells were collected. Multiplex assays were conducted using the Human Procartaplex Mix&Match 8-Plex (Invitrogen) according to the manufacturer’s instructions. Data analysis was conducted using a Bio-Plex 200 system with Bio-Plex Manager software (Bio-Rad).

### Live imaging

Plated macrophages were labeled with 5 µM CellTrace Violet Cell Proliferation Kit (Invitrogen). Activated human CD8 T cells were stained with 5 mM CellTrace CFSE Cell Proliferation Kit (Invitrogen) and added to macrophages at a ratio of 2:1. Interactions were monitored for 2 hours, with acquisitions every 60 s. Imaging was conducted using an upright FVMPE-RS microscope (Olympus) equipped with a 25×/1.05 numerical aperture water-dipping objective (Olympus). Excitation was provided by an InSight DeepSee dual laser (Spectra Physics) set to 830 nm. Fluorescence detection was performed using the following filters: CFP (483/32) and GFP (520/35). Movies were processed and analyzed using Fiji software. Movie analyses were conducted in a blinded manner. Movies and figures are presented as two-dimensional maximum intensity projections derived from 3D data.

### Generation of bone marrow chimeras

Recipient mice (CD45.1^+^ wild-type or IFNAR-deficient) were γ-irradiated with two doses of 5.5 Gy, 4 hours apart. Bone marrow cells were harvested from the femurs and tibias of donor mice (CD45.1^+^ or IFNAR), and single-cell suspensions were prepared by mechanical dissociation and filtration through a 70 µm cell strainer. A total of 0,5×10⁶ donor bone marrow cells were intravenously injected into each irradiated recipient. Chimeric mice were maintained under specific pathogen-free conditions. Reconstitution of the hematopoietic compartment was assessed 10 weeks later by flow cytometry of peripheral blood leucocytes.

### Generation of chimeric antibodies

Anti-PD-1 mAb (clone RMP1-14) V_H_ synthesis in pUC19-hIgγ1.N_297_A or pUC19-hIgγ4. S_228_P, and V_L_ synthesis in pUC-hkappachain-expressing vectors were performed by Synbio Technologies. Antibodies were produced by transient co-transfection of V_H_-C_H_ and V_L_-C_L_ expression plasmids into exponentially growing Freestyle HEK 293-F EXPI cells that were cultured in serum-free Freestyle 293 Expression Medium (Life Technologies) in suspension at 37°C in a humidified 8% CO₂_2_ incubator on a shaker platform rotating at 110 rpm. 24 hours before transfection, cells were harvested by centrifugation at 300 × g for 5 min and resuspended in expression medium at a density of 2×10^6^ cells/mL, and cultured overnight in the same conditions as mentioned above. To produce mAbs, 40 µg of each V_H_ and V_L_ expressing plasmids were diluted in 80 µL of FectoPRO reagent (Polyplus) at a final DNA concentration of 0.8 µg/mL, incubated for 10 min at RT before addition to the cells. 24 hours post-transfection, cells were diluted 1:1 with expression medium. Cells were cultured for 6 days after transfection, supernatants were harvested, centrifuged at 1800 g for 40 min and filtered (0.2 µm). Antibodies were purified by affinity chromatography using an AKTA pure 25L FPLC instrument (GE Healthcare) on a HiTrap Protein A Column (GE Healthcare) and desalted on a HiTrap Desalting Column (GE Healthcare).

### Quantification of cell proliferation

Cell generations were identified using the Proliferation tool in FlowJo V.10.8.1. The replication index was calculated as the average number of daughter cells produced by cells that had divided at least once. The replication index was calculated using the following formula: $Replication\;index\;=\frac{\sum_{1}^{i}N_{i}}{\sum_{1}^{i}\frac{N_{i}}{2^{i}}}$ where *i* is the generation of cells and *N_i_* the number of cells in each generation.^36^

### Statistical analysis

All statistical analyses were conducted using Prism V.9.5.0 (GraphPad). Data are presented as mean±SEM. Given the variability of biological data, we assessed normality and homogeneity of variance before applying the non-parametric tests. Mann-Whitney t-test was used for comparisons between two groups, while Kruskal-Wallis one-way analysis of variance was employed for multiple comparisons. For mice survival, statistical analysis was performed using a log-rank test. All statistical tests were two-tailed with a significance level of 0.05. ∗p<0.05, ∗∗p<0.01, ∗∗∗p<0.001; ns, non-significant.

## Supplementary material

10.1136/jitc-2025-014665 online supplemental file 1

10.1136/jitc-2025-014665 online supplemental file 2

## Data Availability

Data are available on reasonable request.
