# Supplementary material for: Lymph node fine-tuning FcγR signaling boosts anti-PD-1 therapy
Source: J Immunother Cancer. 2026 Jun 12;14(6):e014665. doi: 10.1136/jitc-2025-014665 (PMC13289414; doi:10.1136/jitc-2025-014665)
Supplement: online supplemental file 1 [file jitc-14-6-s001.docx]

**Lymph node fine-tuning FcγR signaling boosts anti-PD-1 therapy**

**Supplemental Material**

**Figure S1. Absence of chemokine production in the serum of anti-PD-1 treated mice.**

C57BL/6 mice were injected s.c. with MC38-OVA tumor cells (0.5.10^6^ cells). After 10 days, mice were treated with anti-PD-1 alone (250 µg, i.v) or with isotype control (250 µg, i.v). Chemokines production was measured in the serum of mice on day 3 post treatment.


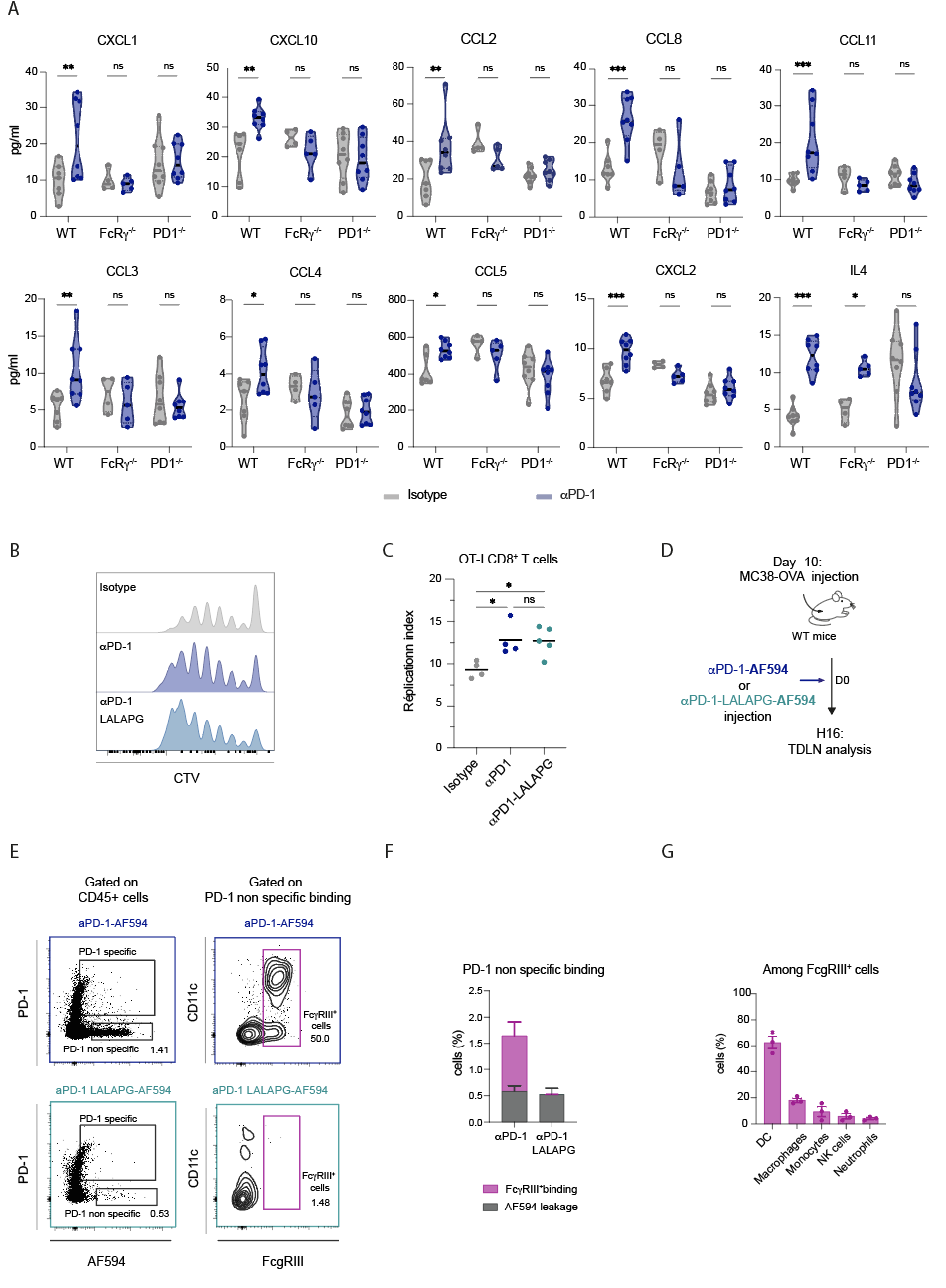


**Figure S2. Both PD-1 and FcγRIII engagement are essential for chemokine production.**

**A**) WT, FcRγ^-/-^ or PD1^-/-^ mice were injected s.c. with MC38-OVA tumor cells (0.5.10^6^ cells). After 10 days, mice were treated with anti-PD-1 (250 µg, i.v) or with isotype control (250 µg, i.v). Chemokines production was measured in the lysate of the tumor-draining lymph node of WT, FcRγ^-/-^, and PD-1^-/-^ mice on day 3 post treatment. Compiled from two independent experiments with 6-8 mice per group. **B-C**) MC38-OVA tumor-bearing mice were adoptively transferred with naïve CTV-labelled OT-I CD8+ T cells on day 10 and treated with anti-PD-1 mAb, anti-PD-1 LALAPG mAb or with isotype control. OT-I CD8+ T cell proliferation in the draining lymph node was assessed on day 3 post treatment. **B**) Representative histograms showing CTV dilution in OT-I CD8+ T cells. **C**) Absolute number of undivided OT-I CD8+ T cells in lymph nodes from mice bearing MC38-OVA. **D-G**) C57BL/6 mice were injected s.c. with MC38-OVA tumor cells. After 10 days, tumor-bearing mice were treated with AF594-labelled anti-PD-1 mAb (250 µg, i.v.), AF594-labelled anti-PD-1 LALAPG mAb or with isotype as a control. Flow cytometric analysis of draining lymph nodes was performed 16 hours later. **D**) Experimental setup. **E**) Representative FACS dot plot showing *in vivo* labelling by AF594-labelled anti-PD-1 WT and LALAPG variant mAb (clone RMP1-14) and *ex vivo* staining by anti-PD-1 (clone 29F.1A12). Region “PD-1 specific” corresponds to cells binding the Ab injected *in vivo* and expressing PD-1 as detected by *ex vivo* staining, region “PD-1 non specific” corresponds to non-specific binding, including FcgR binding and AF594 aspecific binding. **F**) Quantification of PD-1 non specific binding highlights a FcγRIII specific binding in the anti-PD-1 WT variant group. **G**) Proportion of each anti-PD-1 AF594^+^ FcgRIII^+^ myeloid cell subset involved. Statistical analyses were performed using Kruskal–Wallis one-way ANOVA. ns non-significant; *, p<0.05; **, p<0.01, ***, p<0.001.


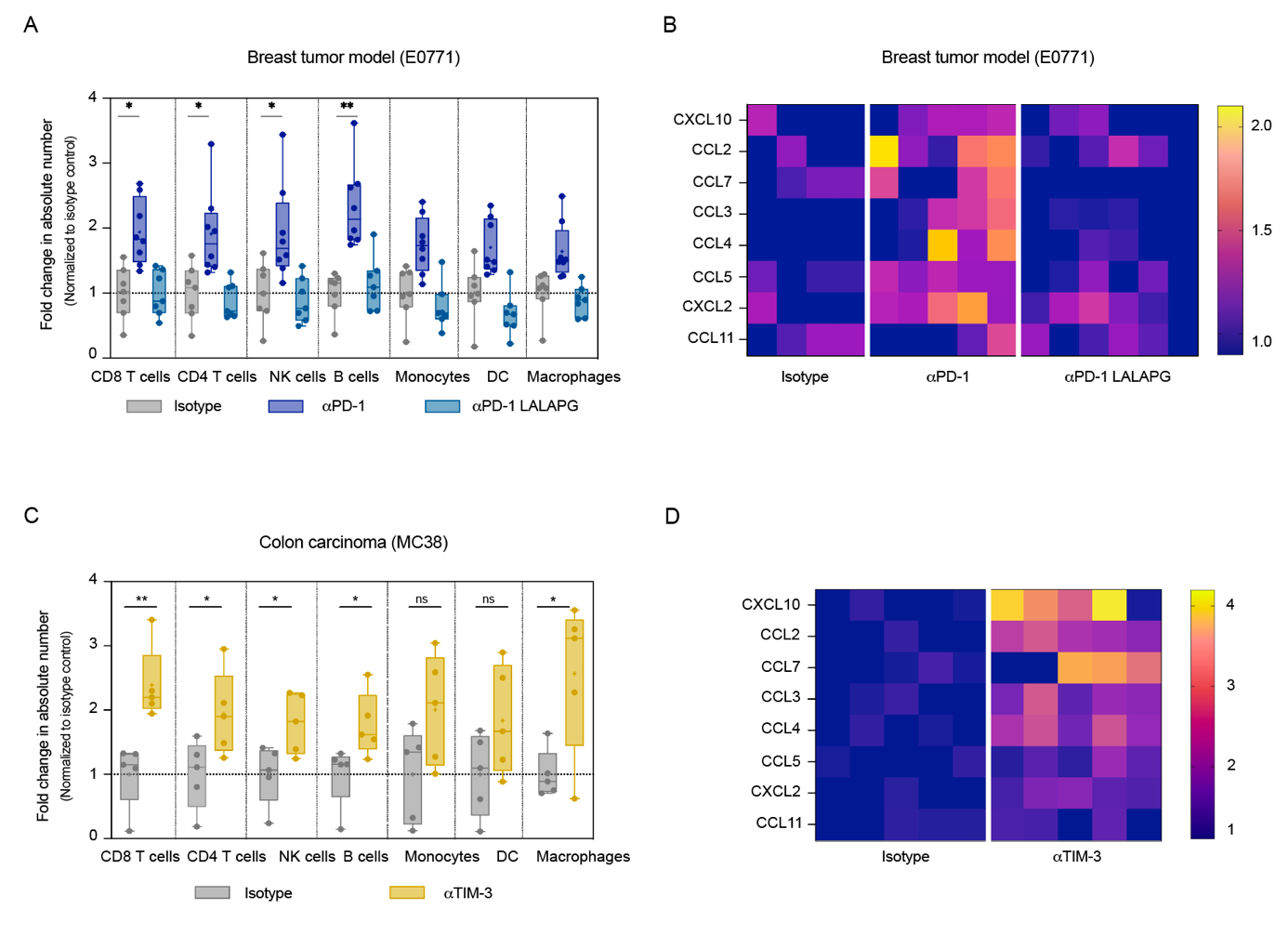


**Figure S3. The FcγR-dependent activity of anti-PD-1 mAb is conserved across tumor models and immune checkpoint inhibitors.**

**A-B**) E0771 breast tumor-bearing mice were treated on day 10 with anti-PD-1 mAb or with isotype control. **A**) Quantification of immune cells was performed 3 days later by flow cytometry in tumor-draining lymph nodes, represented as fold changes in absolute number compared to the isotype control group. Compiled from two independent experiments with a total of 7-8 mice per group. **B**) Heat map showing the cytokine landscape of draining lymph nodes measured by a multiplex protein assay three days after treatment. Values were normalized to the mean value measured for control isotype-treated mice. Compiled from two independent experiments with a total of 5-6 mice per group. **C-D**) MC38-OVA tumor-bearing mice were treated on day 10 with anti-Tim3 mAb or with isotype control. **C**) Quantification of immune cells was performed 3 days later by flow cytometry in tumor-draining lymph nodes, represented as fold changes in absolute number compared to the isotype control group with a total of 5 mice per group. **D**) Heat map showing the cytokine landscape of draining lymph nodes measured by a multiplex protein assay three days after treatment. Values were normalized to the mean value measured for control isotype-treated mice. Compiled from two independent experiments with a total of 5 mice per group. Statistical analyses were performed using Mann–Whitney t-tests. *, p<0.05; **, p<0.01, ***, p<0.001.

**Figure S4. Human macrophages are able to bind the Fc portion of nivolumab.**

Macrophages derived from donor PBMCs were stained with an AF594-labeled isotype control, AF594-labeled nivolumab, or pre-incubated with an Fc blocker prior to staining with AF594-labeled nivolumab. MFI of antibody binding is represented as histogram and bar plot. One representative of two independent experiments.


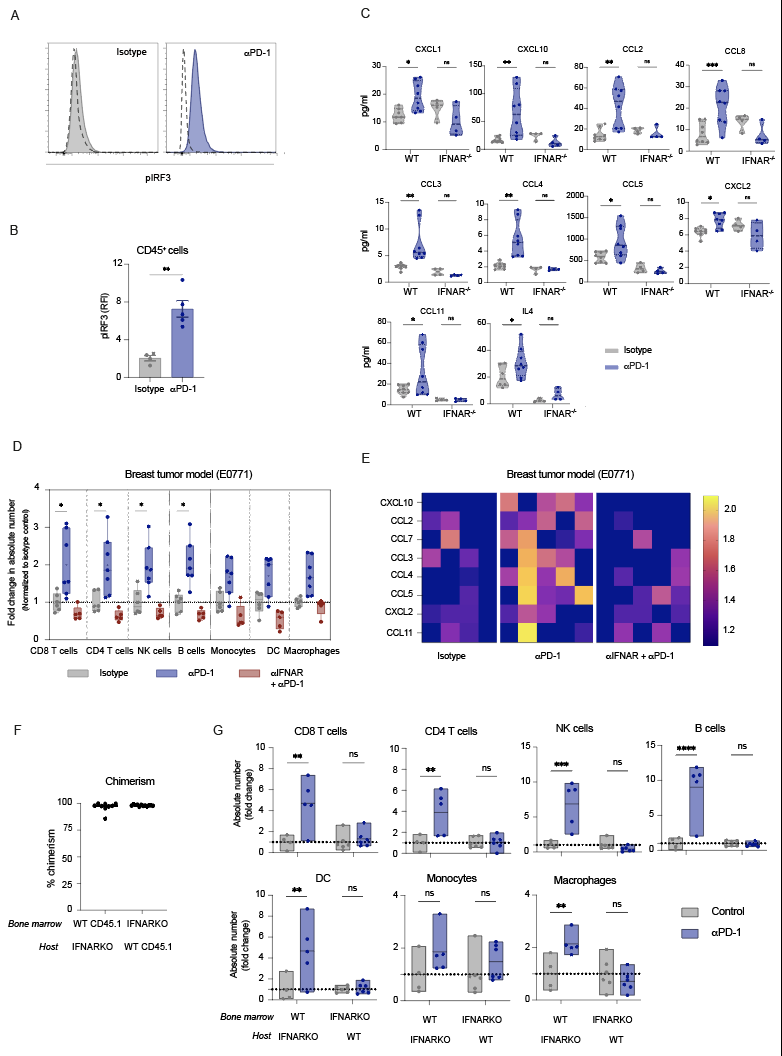


**Figure S5. Type I IFN signaling is essential for chemokine production and immune cell recruitment.**

**A-B)** MC38-OVA tumor-bearing mice were treated on day 10 with anti-PD-1 mAb or with isotype control. Detection of p-IRF3 in CD45^+^ lymph node cells 16 hours after treatment. **A**) Representative histograms showing p-IRF3 staining. **B**) MFI of p-IRF3 staining on CD45^+^ cells normalized to that measured with an isotype control. Representative of two independent experiments with a total of 4-5 mice per group. **C**) WT or IFNAR^-/-^ mice were injected s.c. with MC38-OVA tumor cells (0.5.10^6^ cells). After 10 days, mice were treated with anti-PD-1 (250 µg, i.v) or with isotype control (250 µg, i.v). Chemokines production was measured in the lysate of the tumor-draining lymph node of WT and IFNAR^-/-^ mice on day 3 post treatment. Compiled from two independent experiments with 4-8 mice per group. **D-E**) C57BL/6 mice were injected s.c. with E0771 breast tumor cells. After 10 days mice were treated with anti-PD-1 mAb with or without anti-IFNAR mAb. **D)** Quantification of immune cells was performed 3 days later by flow cytometry in tumor-draining lymph nodes, represented as fold changes in absolute number compared to the isotype control group with a total of 5-7 mice per group. **E)** Heat map showing the cytokine landscape of draining lymph nodes measured by a multiplex protein assay three days after treatment. Values were normalized to the mean value measured for control isotype-treated mice with a total of 4-5 mice per group. **F-G**) CD45.1+ and IFNAR-deficient recipient mice were lethally irradiated and reconstituted with WT (CD45.1^+^) and IFNARKO (CD45.2^+^) bone marrow cells. Chimeric mice were injected intravenously and, 12 weeks later, challenged with MC38-OVA tumors. Ten days after tumor implantation, mice were treated with anti–PD-1 mAb or isotype control. (**F**) Chimerism in the tumor-draining lymph nodes was quantified for each mouse. (**G**) Immune cell populations were analyzed three days later by flow cytometry in the tumor-draining lymph nodes, with results expressed as fold changes in absolute numbers relative to the isotype control group. Statistical analyses were performed using Kruskal–Wallis one-way ANOVA. ns non-significant; *, p<0.05; **, p<0.01, ***, p<0.001.


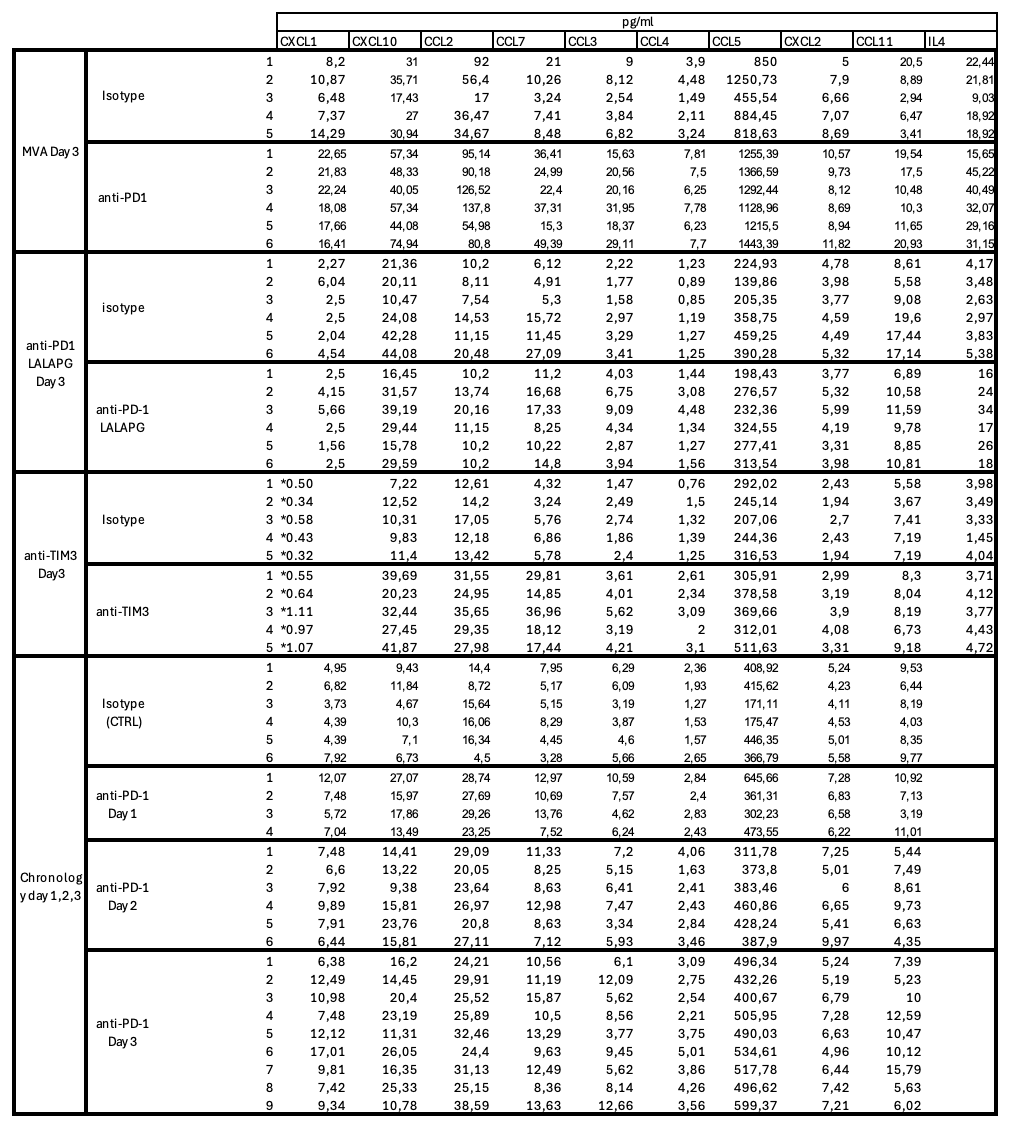


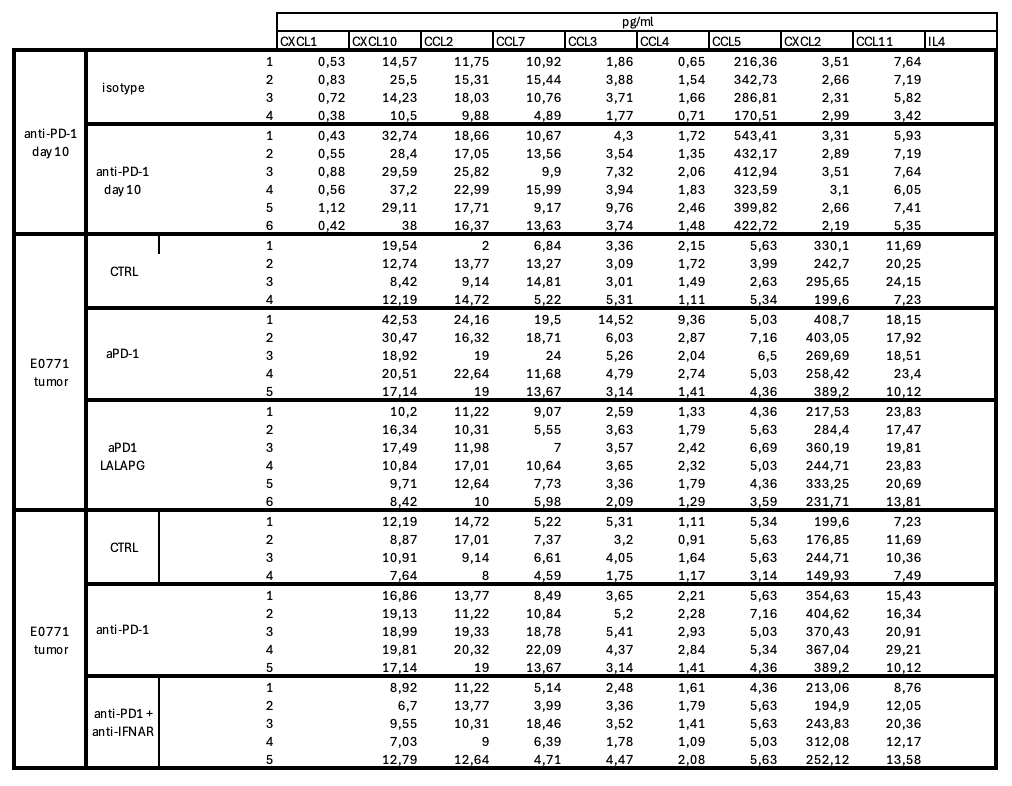


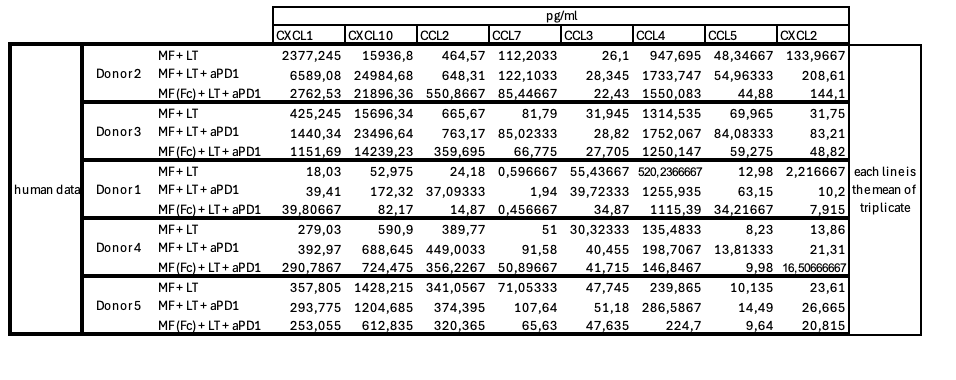


Supplemental Table 1. Raw data concentration of chemokine
